# Oxygen-defective electrostrictors for soft electromechanics

**DOI:** 10.1126/sciadv.adq3444

**Published:** 2024-08-30

**Authors:** Victor Buratto Tinti, Jin Kyu Han, Valdemar Frederiksen, Huaiyu Chen, Jesper Wallentin, Innokenty Kantor, Anton Lyksborg-Andersen, Thomas Willum Hansen, Garam Bae, Wooseok Song, Eugen Stamate, Daniel Zanetti de Florio, Henrik Bruus, Vincenzo Esposito

**Affiliations:** ^1^ Department of Energy Conversion and Storage, Technical University of Denmark, 2800 Kgs. Lyngby, Denmark.; ^2^ Center for Engineering, Modeling and Applied Social Sciences, Federal University of ABC, Santo André 09210-580, SP, Brazil.; ^3^ Institute of Materials Research and Engineering, A*STAR (Agency for Science, Technology and Research), 138634 Singapore, Singapore.; ^4^ Department of Physics, Technical University of Denmark, 2800 Kgs. Lyngby, Denmark.; ^5^ Synchrotron Radiation Research and NanoLund, Lund University, Box 118, Lund 22100, Sweden.; ^6^ MAX IV Laboratory, Lund University, Lund, Sweden.; ^7^ National Centre for Nano Fabrication and Characterization, Technical University of Denmark, 2800 Kgs. Lyngby, Denmark.; ^8^ Thin Film Materials Research Center, Korea Research Institute of Chemical Technology (KRICT), 141 Gajeong-ro, Yuseong-gu, Daejeon 34114, Republic of Korea.; ^9^ School of Electronic and Electrical Engineering, Sungkyunkwan University, Suwon 16149, Republic of Korea.

## Abstract

Electromechanical metal oxides, such as piezoceramics, are often incompatible with soft polymers due to their crystallinity requirements, leading to high processing temperatures. This study explores the potential of ceria-based thin films as electromechanical actuators for flexible electronics. Oxygen-deficient fluorites, like cerium oxide, are centrosymmetric nonpiezoelectric crystalline metal oxides that demonstrate giant electrostriction. These films, deposited at low temperatures, integrate seamlessly with various soft substrates like polyimide and PET. Ceria thin films exhibit remarkable electrostriction (*M*_33_ > 10^−16^ m^2^ V^−2^) and inverse pseudo-piezo coefficients (*e*_33_ > 500 pmV^−1^), enabling large displacements in soft electromechanical systems. Our study explores resonant and off-resonant configurations in the low-frequency regime (<1 kHz), demonstrating versatility for three-dimensional and transparent electronics. This work advances the understanding of oxygen-defective metal oxide electromechanical properties and paves the way for developing versatile and efficient electromechanical systems for applications in biomedical devices, optical devices, and beyond.

## Teaser

Soft materials meet oxygen-defective metal oxide thin films, empowering electromechanical performance for flexible electronics.

## INTRODUCTION

Every dielectric material responds to electric fields by changing shape, a phenomenon known as electrostriction (*1*). Electrostrictors commonly deliver limited strain. They follow Newnham’s empirical quadratic law (*2*), where the electrostriction coefficient is proportional to the mechanical appliance and inversely to the dielectric constant (ε). Therefore, highly polarizable ceramics with high dielectric constant, e.g., PMN-PT relaxor ferroelectrics (ε *>* 10,000), usually achieve high electrostriction, with a typical coefficient (*M_ij_*) in the order of 10^−18^ to 10^−17^ m^2^ V^−2^ (*3*).

Recent findings have revealed that oxygen-defective fluorites such as ceria, zirconia, and bismuth oxide can exhibit notably higher electromechanical responses than predicted by Newnham’s law, surpassing the theoretical values by several orders of magnitude (*4*–*6*). This effect has been defined as nonclassical (*7*) or giant (*4*) electrostriction and is attributed to their unique defect chemistry (*4*, *5*). The large concentration of electronic defects, i.e., oxygen vacancies ( $V_{O}^{\cdot \cdot }$ ), induces a high density of elastic dipoles, which are electrostatically activated under the moderated electric fields (*8*). Notably, cerium oxide and the other fluorites are centrosymmetric (*9*) and thus do not have piezoelectric or ferroelectric properties. However, they demonstrate exceptional electrostriction capabilities, achieving and surpassing those of traditional piezoelectric materials (*4*, *5*, *10*, *11*). As an example, gadolinium-doped cerium oxide (Ce_1−*x*_Gd*_x_*O_2−*x*/2_, CGO) exhibits a relative electrical permittivity of approximately 30 (*5*, *12*), which is considerably lower than relaxor ferroelectrics (*13*, *14*). However, ceria can attain high electromechanical properties with electrostriction coefficient (*M_ii_*) that reach up to 10^−17^ m^2^ V^−2^ in the bulk form (*15*) and 10^−14^ m^2^ V^−2^ in atomically engineered heterostructures (*16*).

An explanation of the underlying mechanics behind the nonclassical electrostriction in the various defective fluorites is still disjointed. However, a commonly accepted theory explains this phenomenon in oxygen-defective ceria. Acceptor-doped ceria, e.g., Gd-doped ceria (CGO), has high oxygen deficiency ($V_{O}^{\cdot \cdot }$) from the different oxidation states of the cations, i.e., Ce^4+^, Ce^3+^, and Gd^3+^. Atomic Ce^3+^ defects are created due to electrons released due $V_{o}^{\cdot \cdot }$ formation ( $O_{O}^{\times }\to 1/2O_{2}+V_{o}^{\cdot \cdot }+2e\prime$ ), being incorporated by nearby Ce^4+^ cations. The oxygen defects induce viscoelastic distortion due to the singularities of the lattice’s electrostatic and elastic forces (*4*, *17*). Density functional theory simulations predict that atomic distortion generates orientable elastic dipoles, i.e., change of local oxidation and bond length, that instantaneously polarize under the applied electric fields (*18*, *19*).

One important feature of nonclassical electrostrictors is that the cubic structure applies to polycrystalline thin films without requiring specific orientation. Unlike ferroelectric piezoelectrics, there is no need for prior electrical polarization, as this feature is intrinsic/extrinsic and defect-induced (*5*). In addition, ceria electrostrictors do not require high crystallinity to exhibit high electromechanical performance but instead thrive in nanometric systems (*20*). Conversely, piezoceramics necessitate high crystallinity, imposing limitations on preparation methods, often involving high processing temperatures (*21*). The high temperature required when working with piezoceramics severely restricts their compatibility with temperature-sensitive materials, such as the polymers used in flexible electronics (*22*), forcing complex thin-film transfer and buffering methods (*20*, *22*).

The versatility of ceria-based actuators potentially enables deposition at room temperature (*20*), making them compatible with a wide range of low-temperature flexible substrates, e.g., polymers. Ceria actuators would allow the direct deposition of active electromechanical materials on low-temperature substrates, further advancing this technology for impactful soft integrated micro-actuators (*23*).

Last, as for any electrostrictor, ceria’s electromechanical response is proportional to the square of the applied electric field (*1*). Therefore, the electrostriction coefficients tensor (*M_ij_*) is used as the scalar of the square of the electric field to generate observed strain. However, if a bias (*U*_DC_) is applied in conjunction with an alternating field (*U*_AC_), a large pseudo-piezoelectric response can be induced (*2*), i.e., the first-harmonic oscillation linear to the applied electric field (*10*). Such a feature further enhances the versatility of ceria actuators, mimicking the response of current piezoceramics, defining the so-called pseudo-piezo coefficients, *e_ij_* (see the Supplementary Materials and Materials and Methods for details) (*4*, *10*).

This work proposes an approach for developing micro-electromechanical systems (MEMS) with oxygen-defective electrostrictors directly integrated as thin films into flexible substrates and soft polymers. The concept’s versatility is proved in various actuating configurations with high technological relevance, including transparent substrates, optical media, and three-dimensional (3D) shapes. Nonclassical CGO electrostrictors were chosen for the material’s relatively known properties and deposition parameters (*18*). We used the radio frequency magnetron sputtering (RF-MS) deposition technique to deposit our ceramic thin films, a straightforward physical vapor deposition (PVD) technique widely used in several thin-film technologies. The RF-MS technique is suitable for near–room temperature deposition onto flexible electronics (*24*, *25*).

## RESULTS

### Electromechanical, structural, and microstructural features

Because of its favorable mechanical and chemical stability properties, we chose polyimide (PI) as our reference substrate for investigating flexible substrates. PI is also a key polymer in flexible electronics (*26*) and medicine, e.g., implantable (*27*). With Young’s modulus of approximately 4 GPa and tensile strength of around 80 MPa (*28*), PI contributes to its suitability as a solid and easily manipulable substrate. Furthermore, substrates are readily available in various dimensions and have already been used in developing MEMS (*29*). PI is also ideal for initial testing due to its high heat resistance. Although the film deposition is conducted without intentional substrate heating, plasma-based deposition processes can induce mild heating (>120°C), which can cause softening in temperature-sensitive polymers (*30*). PI’s thermal stability is desirable for the initial test but not strictly necessary, as we could also sputter the ceramic films onto less stable polymers, such as polyethylene terephthalate (PET). To illustrate the capabilities of a flexible substrate, we also used a ceramic substrate to enable comparative analysis in this work. Thin films were thus deposited under the same deposition conditions on glass substrates, i.e., fused silica (FS). Silica glasses are well established and widely used, providing a reliable benchmark for stiff substrates (*30*, *31*).

Previous work indicates that crystallinity and disorder embedded in nanocrystalline structures in polycrystalline ceramics do not suppress the electromechanical properties of polycrystalline doped ceria (*32*, *33*). PVD methods at low temperatures are also known to yield crystalline disorder due to the low diffusivity of the chemical species on a cold substrate (*34*). In such conditions, while the energy and chemical species in the plasma remain critical, the chemical and physical properties of the substrate result in less relevant influence on the nucleation and growth of the deposited film (*34*, *35*).

**Fig. 1.**
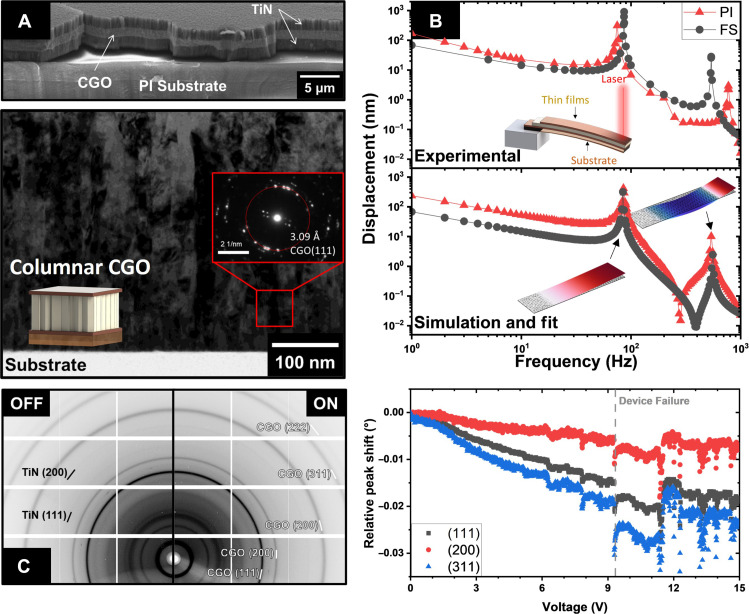
Flat cantilevers. (**A**) Cross-section view by scanning electron microscopy (SEM) and TEM of the columnar microstructure of the CGO and TiN thin films. (**B**) Experimental and simulated electrostrictive responses of cantilevers based on ceria thin films (ca. 500 nm) on polyimide (PI) and fused silica (FS) substrates. The bias (*U*_DC_, 60 kV/cm) that induced first-harmonic response (pseudo-piezoelectric) was measured at 10 Hz under a 6-kV alternating field. (**C**) ioT-XRD of the as-prepared and under-bias films. The sample was kept under a 0.4 MV/cm bias for 12 hours for the “ON” state. The right graph shows the (111), (200), and (311) peak shifts with increasing bias from 15 V at a rate of 0.1 V/s.

Figure 1A illustrates various typical features of the CGO thin films deposited on PI with a TiN bottom electrode as a typical example of the microstructures synthesized by RF-MS at room temperature (without intentional substrate heating). The TiN coating can effectively adhere to a wide range of polymers and is compatible with sputtering techniques (*36*, *37*). TiN is also an excellent electronic conductor with a typical 1 to 100 S cm^−1^ conductivity. Previous investigations showed that TiN is a promising layer for overcoming the adhesion problem between oxide films and silicon and excels in electromechanical applications based on ceria, producing durable and reliable electromechanical devices compared to metal-based electrodes (*38*).

All samples maintain similar crystalline characteristics regardless of the substrates, whether glass, PI, or PET (fig. S1). Both CGO and TiN films were found to be polycrystalline and displayed relatively broad diffraction peaks. X-ray diffraction (XRD) patterns of CGO and TiN thin films match the fluorite structure ( $\text{Fm}\bar{3}m$ ). No evident differences in substrate or secondary phases could be identified among all samples.

Further details of the CGO thin-film microstructure can be seen in the images by bright-field TEM. The analysis confirms columnar grain growth. Similar microstructures have been previously reported for ceria thin films deposited by sputtering (*20*, *39*). Such a feature is usually attributed to the directional nature of the sputtering process. The limited mobility imposed by low substrate temperature coupled with seeds formed during the initial deposition stages contributes to the formation of such microstructure (*40*, *41*). Electron diffraction patterns confirm the presence of a crystalline cubic structure, matching planar distances to ceria. The analysis also indicates a high level of crystalline disorder in the structure.

The electromechanical properties are characterized by the one-end fixed cantilever configuration. Such a geometry was chosen because it facilitates the conversion from the local cantilever deflection into stress and the electromechanical coefficients with high precision (see Materials and Methods). Figure 1B shows the frequency-dependent electromechanical response of the CGO10 films deposited on glass and PI substrates in the cantilever configuration. The electromechanical properties of CGO in the *M*_31_ mode are characterized using TiN as the bottom and top electrodes. Both samples on PI and glass show similar electromechanical features, achieving displacements in several tens of nanometers. Using Stoney’s formalism, we can promptly calculate the electromechanical performance of the PI and glass cantilevers (see Materials and Methods). Both samples achieved *M*_31_ around ≈ 10^−19^ m^2^ V^−2^ and *e*_31_ ≈ 5 pm V^−1^ (under a 60 kV/cm bias) (*10*, *42*). Such values are comparable to those previously reported for ceria-based thin films. The *M*_31_ coefficient tends to be lower when compared to *M*_33_, which is usually between 10^−18^ and 10^−16^ m^2^ V^−2^ (*4*, *12*, *43*).

The samples on PI and glass exhibited displacement peaks around 85 and 550 Hz, attributed to the cantilevers’ first and second resonance vibrational modes (Fig. 1B). The overall behavior of the cantilever is successfully simulated and fitted using finite elements simulations. The simulations confirm the resonance modes. The difference in peak frequency and intensity is attributed to variations in the cantilevers’ mass, dimensions, and mechanical properties (see the Supplementary Materials for the details). The simulation indicates that the *M*_33_ is the dominant actuation mode, even with a cross-plane geometry. The reason for this is illustrated in Fig. 1B (detailed in figs. S2 and S3), which shows the cross section of the cantilever device near the edge of the top electrode. In the large region of length *L*_te_ beneath the top electrode, the electric field is perpendicular to the CGO film, which promotes the transverse *M*_31_-coupling. However, in a small region of length *h* approximately equal to the thickness of the CGO film, the electric (fringe) field acquires a substantial component parallel to the CGO film, which promotes the longitudinal *M*_33_-coupling. This coupling becomes dominant if *M*_33_ *h* is larger than *M*_31_ *L*_te_.

The polymer-based cantilever exhibited an electromechanical response five times higher at low frequencies (<100 Hz) than the sample deposited on glass. However, the performance of the polymer-based cantilever drops quickly for frequencies above 100 Hz, exhibiting lower displacement than the ceramic substrate. Such an effect is due to the PI substrate’s viscoelastic properties, leading to severe attenuation due to dampening, which becomes progressively prominent with frequency (*44*).

In operando synchrotron transmission XRD (ioT-XRD) analyses were carried out to obtain a more comprehensive understanding of the crystalline structure of the deposited CGO films. The ioT-XRD results (Figure 1C) confirmed the polycrystalline nature of the sputtered thin films. No texture could be observed, which is expected for films deposited at room temperature due to the limited atomic mobility. Despite the high resolution and intensity of the signals of the synchrotron line (see Materials and Methods), the in operando measurements (Fig. 1C and fig. S4) show no clear change to the diffraction pattern upon applying an electric field. Such results emphasize the stability of ceria’s fluorite structure and the TiN electrodes. Notably, we did not observe the formation of any cerium oxide tetragonal phase in operando previously claimed in polycrystalline CGO deposited at high temperatures as columnar crystalline thin films (*45*).

Although no changes to the diffraction pattern due to the applied bias of ceria can be visually identified, careful refinement of the results unveils a clear electromechanical response at the structure (fig. S4). With an increase in voltage, there is a clear shift of the diffraction peaks to lower angles (Fig. 1C), indicating an expansion of planar distances and the unit cell. Since the electric field is being applied out-of-plane along the [111] direction and the diffraction experiment is in the transmission geometry, an observed expansion is expected due to the positive *M*_31_ coefficient associated with the electrostrictive behaviour. Notably, the expansion of the cell along the [111] direction is larger than for the [100]. This observed expansion marks the first reported instance of crystalline structural strain due to an electric field for electromechanically active ceria-based ceramics. Previous reports indicate that ceria can be reduced under a DC electric field (*46*), which can induce an overall expansion of the unit cell due to the reduction of Ce^4+^ to Ce^3+^ (*47*).

During stability ioT-XRD measurements (12 hours) under intense electric bias (0.4 MV/cm), we observed other effects that could be associated with electromigration and electrochemical reactions between the CGO and electrodes (fig. S1). Nevertheless, the films showed chemical and mechanical stability under alternating electric fields above 10 kV/cm for more than 10^3^ cycles with no signs of degradation (see stability test in fig. S1).

In summary, ceria-based electromechanically active thin films were shown to be suitable for integration with PI. Low-temperature processing was capable of producing highly active but sufficiently stable films while being essential for integration with temperature-sensitive polymers. Our results also indicate an electric field-induced crystalline overall anisotropic expansion of the films.

### 3D deposition: Microtubes

To further develop the concept of coupling flexible substrates and ceria actuators, we extended the development demonstrated for flat films to 3D shapes by using microcapillaries as substrates. The tubular geometry is highly relevant for biomedical applications, as it can be used for the creation of a wide range of devices, such as micropumps (*48*), fluid separators (*49*), and reactors (*50*). In addition, the round geometry of a capillary offers an exposed continuous surface, which is ideal for the deposition of thin films using PVD techniques.

**Fig. 2.**
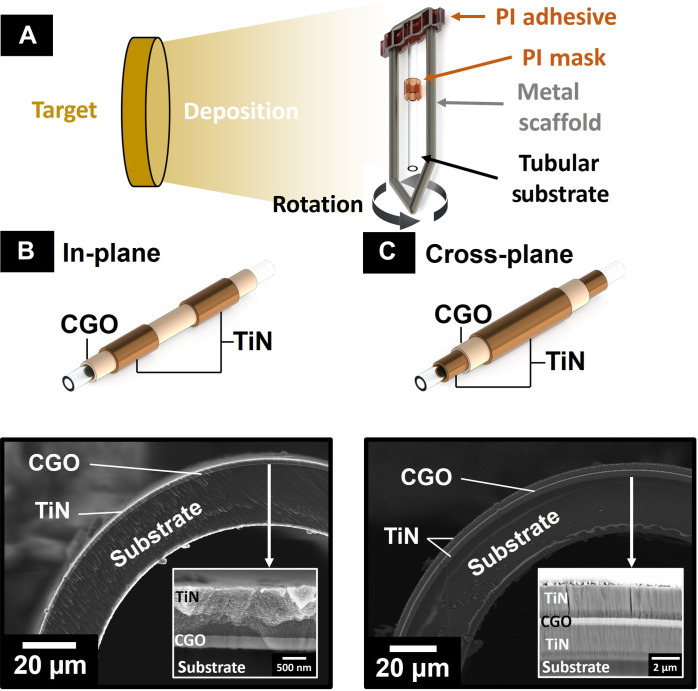
Tubular film coating. (**A**) Illustration of the coating process on the tubular substrates. Cross-section SEM view of the thin films deposited in (**B**) in-plane and (**C**) cross-plane geometries.

Figure 2A depicts the deposition of CGO10 and TiN on a PI tube. Successful deposition of the ceramic films on the tubular devices is evident, not only on the top surface of the tube but also along the sides (fig. S5). The morphology and film thickness of the CGO10 and TiN films at the top of the tube closely resemble the films deposited on flat substrates (Fig. 1B). If taking small-angle steps, the top part of the tube can be considered close to a flat deposition geometry. Further details on the tubular film morphology can be found in the Supplementary Materials.

Figure 2B illustrates the cross section of a tube exposed to a single CGO and TiN deposition without moving the substrate. As a result of only part of the substrate being directly exposed to the target, a thicker film layer forms on one side of the tube, resulting in an asymmetric electromechanical actuator. This asymmetric configuration enables a bending motion similar to a cantilever (*10*). However, such asymmetry can only be used for in-plane electrode configuration. Substantial variation in film thickness and incremental porosity over the surface angle render a cross-plane architecture vulnerable to shorting between top and bottom electrodes. Moreover, an asymmetric deposition would induce deviation from the center axis movement when strain is applied.

Multiple depositions with varying substrate angles were conducted to address these challenges, ensure a homogenous film thickness, and prevent electrode shorting. As the single-layer sample demonstrated that the thickness at 90° was approximately halved, two consecutive depositions of each material (CGO and TiN) were performed with a 180° substrate rotation. The results of double-layer deposition for the cross-plane geometry are presented in Fig. 2C.

Moreover, fig. S5 illustrates the combined film thickness for the cross-plane configuration with double-layered depositions. As expected, we observed a decrease in film thickness moving away from the position directly exposed to the target. Compared to the single deposition, where the thickness dropped to half at 90°, the double-layered sample exhibits a much smaller variation, with only a 10 to 15% decrease at perpendicular positions. Since the fluctuation is symmetrical, it does not adversely affect the application of strain or stress along the tube axis.

**Fig. 3.**
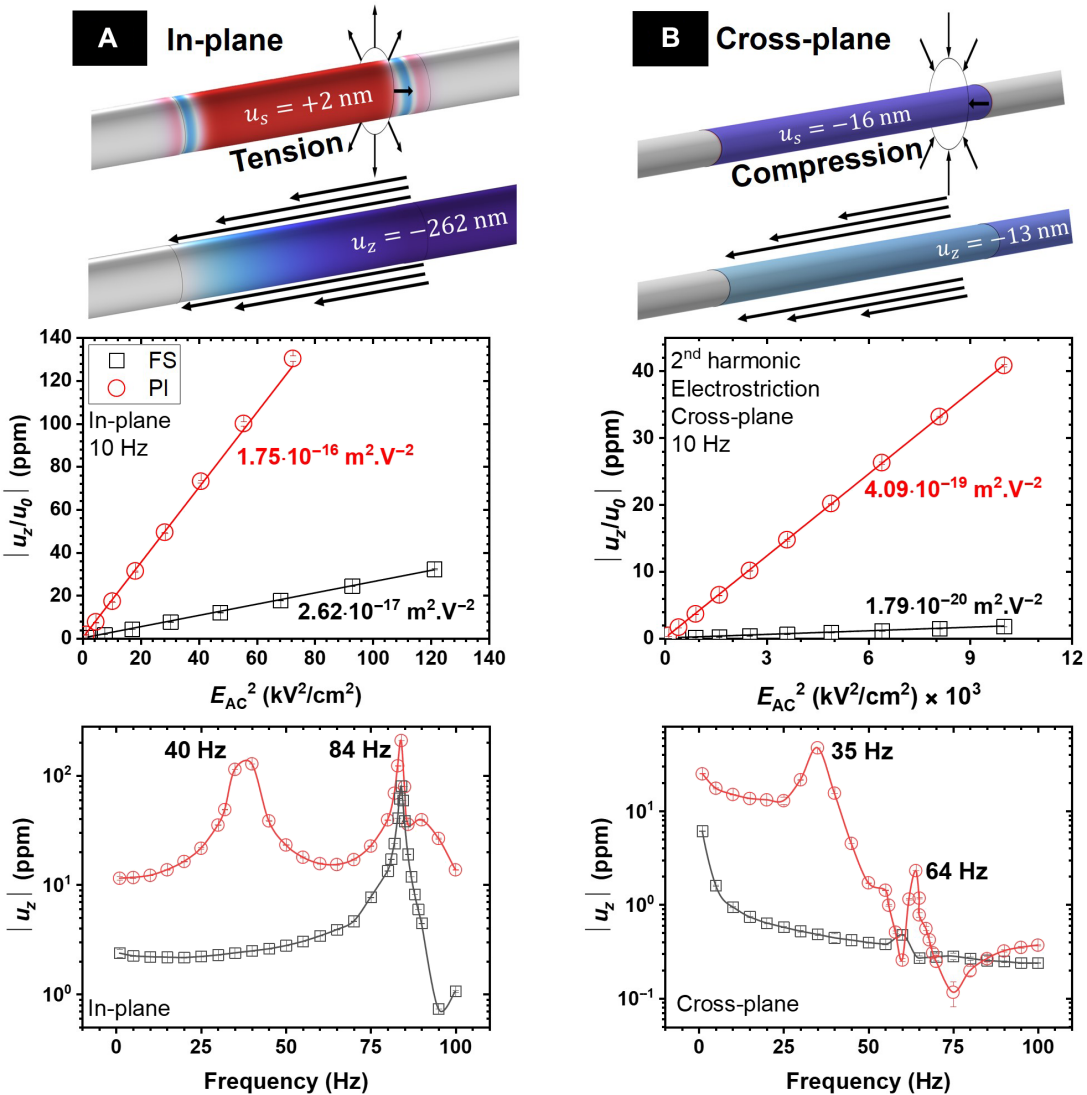
Tubular devices performance. Electromechanical response over field and frequency for the (**A**) in-plane and (**B**) cross-plane tubular devices. The top illustrations are finite element simulations of the geometries at 10 Hz, assuming an *M*_33_ of 6.10^−16^ m^2^/V^2^. The *u_s_* and *u_z_* are relative to the radial and axis displacement.

The double deposition process was thus used to achieve symmetrical devices. Symmetric films maximize elongation/contraction along the tube axis while minimizing side movements. The electrostriction coefficients (*M*_31_ and *M*_33_) values shown in Fig. 3 for the tubes are relative to the overall architecture, including the substrate, rather than solely the CGO film. This approach highlights the impact of the substrate on the final electromechanical response. Detailed information about the device and material performances can be found in Table 1.

**Table 1.** Electromechanical coefficients. Electrostrictive and pseudo-piezoelectric coefficients of the tubular devices were measured at 10 Hz. The device data represent the tube’s performance, including the substrate strain. The specific thin-film performance coefficients can be found below.

@ 10 Hz	Quartz in-plane	PI in-plane	Quartz cross-plane	PI cross-plane
**Device**				
Strain (ppm)*	32.3(2)	156(2)	1.79(1)	40.8(2)
∣*M*_33_∣ (m^2^ V^−2^)	2.51(5)0.10^−17^	1.71(3)0.10^−16^	-	-
∣*e*_33_∣ (pm V^−1^)**	146(1)	724(9)	-	-
∣*M*_31_∣ (m^2^ V^−2^)	-	-	1.7(1)0.10^−20^	4.06(4)0.10^−19^
∣*e*_31_∣ (pm V^−1^)**	-	-	0.41(1)	9.61(7)
**Thin film**				
Stress (MPa)*	42.4(3)	20.9(2)	2.35(1)	5.48(2)
∣*M*_33_∣ (m^2^ V^−2^)	1.65(4)0.10^−16^	1.15(2)0.10^−16^	-	-
∣*e*_33_∣ (pm V^−1^)**	957(7)	486(6)	-	-
∣*M*_31_∣ (m^2^ V^−2^)	-	-	1.09(8)0.10^−19^	2.72(3)0.10^−19^
∣*e*_31_∣ (pm V^−1^)**	-	-	2.7(1)	6.44(5)

*Measured at 10 kV/cm for in-plane and 100 kV/cm for cross-plane.

**Measured with a 10 kV/cm bias for in-plane and 60 kV/cm bias for cross-plane.

Figure 3A illustrates the electrostrictive response (second harmonic) measured parallel to the tube axis (elongation/contraction) for the symmetrical in-plane configuration. The PI-based device exhibits an electrostrictive response approximately one order of magnitude higher than the silica-based device due to the difference in Young’s modulus of the substrates. The elastic modulus of PI is around 4 GPa, while FS has an elastic modulus approximately one order of magnitude higher, around 70 GPa. As a result, the PI substrate imposes less resistance, leading to a higher strain than the glass substrate.

Figure 3B illustrates the electromechanical response (second harmonic) for the symmetrical cross-plane devices. In the cross-plane geometry, the measured strain is perpendicular to the applied field, and thus, the appropriate coefficient to consider is *M*_31_. It is important to note that the *M*_31_ coefficient for ceria is known to be lower compared to the *M*_33_ coefficient (*4*, *10*, *38*). The cross-plane device on PI also showed an improved strain response compared to the ceramic tube.

One noteworthy point for the cross-plane is the proximity between the electrodes and the dielectric strength of the ceria film. In general, voltages up to 800 V can be applied to pellets or in-plane devices without the risk of damaging the sample. However, in the cross-plane geometry, the film separating the electrodes has only a few hundred nanometers (≈500 nm). Thus, only tens of volts can result in extremely high electric fields, and if not careful, can exceed the dielectric strength of ceria (4.5 MV cm^−1^) (*51*). However, this is not necessarily a negative feature, as high electric fields and electromechanical strain/stress can be attained with low voltages (<10 V) (*52*).

For the cross-plane device (Fig. 3B), an apparent relaxation phenomenon is observed at low frequencies (<10 Hz), which is typical for CGO electrostrictors. This behavior was not observed for the in-plane devices, although they were prepared under the same deposition conditions. A possible electro-chemo-mechanical effect at the ceria-electrode interface could contribute to the low-frequency relaxation, as previously reported in similar systems (*53*). The effect is more pronounced at low frequencies (<10 Hz) due to the low kinetics associated with oxygen transfer between ceria and the electrode. The cross-plane configuration, with a larger active CGO-TiN area, may enhance this effect compared to the in-plane devices. Such electro-chemo-mechanical response could occur due to a native TiO_2_ layer formed during deposition (*54*). Redox at the ceria interface with other oxides has been reported previously, and it can generate chemo-mechanical effects, especially in low-frequency regimes where electrochemical processes are dominant (*53*). Yet, the formation of intermediary oxide phases in TiN, such as TiON, has also been previously reported (*55*).

Comparing the response over frequency of the cross-plane and in-plane devices, peaks in the electromechanical response are also visible in Fig. 3B. Similar behavior observed for different electrode geometries indicates that these features arise from the mechanical resonance of the substrate. Simulations could not identify pure resonance modes around this frequency range associated with the tube’s elongation motion. The observed peaks are probably due to lateral instabilities induced by geometrical asymmetries in the samples.

In addition, upon applying a bias, these devices can show an actuation similar to piezoceramics, i.e., first-harmonic response linear to the electric field (fig. S6). Such response can be induced in all devices, reaching a pseudo-piezoelectric coefficient (*e*_33_) of 724 pm V^−1^ (10 kV/cm bias) for the PI-based device. This pseudo-piezoelectric response is instrumental in translating ceria actuators to the current piezoelectric technology, allowing a seamless technology substitution. It also provides a benchmark value for easier performance comparison. The pseudo-piezoelectric response is proportional to the electrostrictive coefficients (*M*_31_ and *M*_33_), and the detailed results can be found in fig. S6 and Table 1.

Asymmetric film depositions were also used to verify if the tubular devices could be actuated in a motion similar to a cantilever. For that, the TiN and CGO10 were only deposited on one side of the tube (without rotating the substrate), similar to the deposition shown in Fig. 2A. A thicker film was created on the substrate’s surface directly exposed to the target, enabling lateral movement.

For completeness, fig. S7 presents the electromechanical response of the asymmetric in-plane devices measured in a cantilever configuration. Both devices were successfully actuated, generating a lateral displacement in tens of nanometers. Calculating the strain/stress induced by the thin films on the substrate is challenging due to the substrates’ complex geometry and the film deposition asymmetry.

Overall, the results indicate that various factors, including film deposition, substrate properties, device geometry, and dimensional variations, influence the electromechanical response and behavior of the tubular shape. Nevertheless, the concentric deposition of thin films proved possible, creating active devices with different geometries and actuation. Using a flexible capillary (PI) enhanced the delivered displacement by more than five times.

### Transparent substrates for optical applications

Transparent electromechanics, e.g., piezo-phototronics and piezo-photonics (*56*), are thriving in metamaterials and advanced optical devices, where the strain at the transparent media drives marked changes in the optical properties of the lenses (*57*). However, these devices are usually complex to be integrated into polymers, as the piezoelectric actuation requires a high level of crystallinity and even optical transparency. Typically, optical-active devices based on piezoceramics are assembled as separate components, complicating the manufacture of micrometric lenses (*58*, *59*). Room temperature ceria direct deposition can thus allow a broader integration of materials and ease of design miniaturization. We conducted tests using transparent PET substrates to validate the concept of flexible devices using CGO electrostrictors.

**Fig. 4.**
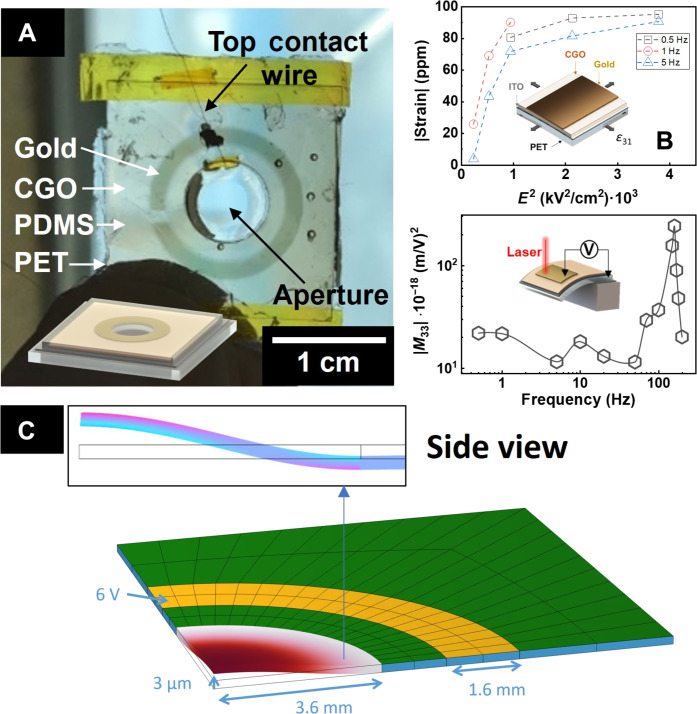
Depositions on transparent PET. (**A**) Photo and schematic representation of the optical device with a centered optical path. (**B**) Electromechanical performance over field and frequency for the sample deposited on ITO/PET. (**C**) Finite element simulations of the proposed geometry under a 3 V.

CGO was directly deposited on an indium tin oxide (ITO)/PET substrate to align the technology to possible optical applications. In the case of creating the lens, ITO was chosen as the electrode material instead of TiN due to its favorable optical properties. Unlike TiN, which is opaque, ITO is transparent to visible light, making it suitable for creating an optical device (Fig. 4A). In addition, ITO has demonstrated compatibility with ceria thin films (*60*), further supporting its selection as the ideal candidate for our lens fabrication.

Figure 4B demonstrates the film orthogonal expansion response induced by cross-plane electrodes. In-plane stress is relevant for optical applications since it causes a curvature in the substrate. Such geometrical changes can be effectively used in tuning the geometry of a lens and, consequently, its focus point. The additional characterization in fig. S8 illustrates the in-plane electromechanical response of CGO grown on ITO/PET substrates. The strain observed for the sample deposited on glass is consistent with previous reports on CGO (*10*, *12*). Notably, for the sample deposited on PET, the electrostriction coefficient achieves a value of 2.3·10^−16^ m^2^ V^−2^ at 1 Hz, which is comparable to the previously shown values (Table 1) and in the high end of what is usually reported for ceria-based actuators (*4*, *61*).

The CGO-PET lens demonstrated a resonant frequency close to 105 Hz (Fig. 4B). The observed resonant phenomenon exceeds the in-plane measurement’s magnitude by more than one order of magnitude. Notably, the effect was enough to visually sense the vibration upon applying an electric field. The calculated stress at 10 Hz was approximately 21 MPa at 30 kV cm^−1^. This effect, coupled with the resonance frequency, allows the electrostriction characteristics to be applied to transparent flexible devices such as optical lenses.

To create our proof-of-concept active lens, we deposited ring-shaped gold top electrodes using a shadow mask (Fig. 4A). The aperture was then immersed in transparent polydimethylsiloxane (PDMS), filling the middle hole and encapsulating the device from the external environment (*62*). Figure 4C describes the electromechanical activation of the device through the CGO ring. The simulations show that we should expect a maximum displacement of 3 μm at the center of the aperture.

An alternating electric field of 50 kV/cm (equivalent to 6 V) was applied for 30 s at the resonant frequency (105 Hz). White light scattering resulting from the roughness of the polymer layer was captured before actuation, with the electric field, and after the test. Figure S9 shows optical microscopy frames captured during the actuation at 50 and 105 Hz. The resonance effect is evident, displaying periodic and uniform motion. This observation supports the result that a considerable strain occurs at the resonance frequency, even though the displacement generated by the CGO ring is only a few nanometers.

**Fig. 5.**
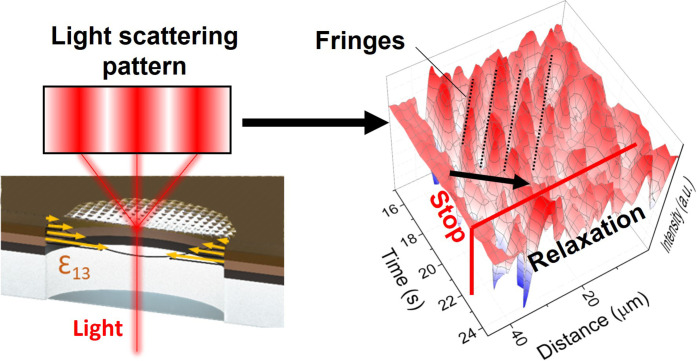
Proof-of-concept lens. Light scattering schematic and 3D graph of the light intensity fluctuations over time at different positions with an excitation at 170 Hz (resonance frequency). Intensity fluctuations are normalized from minimum (0, blue) to maximum (1, red).

Figure 5 presents a 3D view of the wave behavior over time, i.e., based on the data in fig. S9. The video of the effect is also reported as movie S1. The graph depicts a diagonal wave motion occurring after 16 s, followed by the relaxation of the PDMS after removing the electric field. Various waves propagate in different directions, as indicated by the dashed lines. The viscoelastic nature of the PDMS lens leads to circular waves generated within the ring-shaped electrostrictor, creating interference between the waves. The velocity of these waves is related to the elastic constant and density of the polymer (*63*).

Polymer pulse propagation in this frequency range (<20 kHz) has been previously observed (*64*). The longitudinal wave, propagating in the same direction as the electrostrictive resonance vibration of the CGO electrostriction under the PDMS, is transmitted to the upper surface of the PDMS, with some attenuation occurring during this process (*65*). The superposition of multiple waves with time suggests that waves with different starting points and velocities generate interference. This effect supports the occurrence of resonance oscillations. Furthermore, the surface recovers its original position when the electric field is removed, indicating the reversibility and cyclical nature of the effect.

## DISCUSSION

Our study sheds light on how the properties of materials, the design of devices, and their potential applications intersect in flexible electromechanical systems using ceria actuators. We demonstrated their compatibility with various substrate materials, showcasing their versatility in accommodating different design requirements. Notably, we underscore the critical role of functional electrode layers, shedding light on the inadequacies of metallic materials regarding flexibility and compatibility with flexible substrates.

We found that nanocrystalline structures in ceria actuators do not just maintain but improve device performance. This effect contrasts with recent results on coherent films (*61*), which shows that higher degrees of crystallinity can lead to comparable or even better performances (*20*). The improved performance in nanocrystalline ceria actuators likely comes from defects, grain boundaries, and better coupling between electrical and mechanical properties (*33*, *66*).

We also disclose that the material undergoes an overall expansion. However, as expected, the *M*_31_ coefficient is orders of magnitude lower than the *M*_33_ (*4*, *10*, *38*). The simulations pointed out that a considerable amount of the response observed in the transverse mode could be arising from localized parallel mode activation. The *M*_33_ generates substantial stress at the edges of the electrodes due to the electric field alignment change. Such observation indicates that the *M*_31_ for ceria is substantially lower than the reported, possibly following Newnham’s relationship for electrostrictors (*2*). This observation points us to rethink the current magnitude of the transversal mode since parallel mode perturbations are usually deemed negligible for most materials during characterization. In the case of ceria actuators, the several orders of magnitude difference between *M*_33_ (≈10^−16^ m^2^ V^−2^) and the reported *M*_31_ (≈10^−19^ m^2^ V^−2^) make the isolation of pure transverse actuation challenging.

The disparity between the *M*_33_ and *M*_31_ coefficients in ceria actuators presents opportunities for novel device architectures. For instance, the combination of offset cross-plane electrodes, which would typically counteract each other’s effects in piezoceramics and other actuators, becomes feasible and potentially advantageous in ceria-based systems. This unique characteristic allows for actuating high-ratio geometries with reduced voltages, as the opposing modes do not cancel each other due to the overwhelming dominance of *M*_33_. Such unexplored geometries open avenues for designing and optimizing flexible electromechanical systems, offering exciting prospects for future applications in diverse fields such as robotics, biomedical devices, and beyond.

Our investigation highlights the distinct response of ceria-based actuators to various stress configurations. We can finely tune and control strain in flexible devices by harnessing in-plane stress, leading to tailored mechanical behavior. This capability holds the potential for developing advanced flexible electronics and optoelectronics, where precise control over mechanical properties is essential for optimizing device performance and functionality. In addition, manipulating strain through in-plane stress opens up avenues for innovative design strategies and novel applications in fields such as wearable technology, soft robotics, and adaptive structures. Yet, by delving into the diverse shapes and configurations, ceria reveals remarkable adaptability across various applications.

Furthermore, we addressed the distinctions between systems operating at resonance and those operating off-resonance, elucidating the trade-offs associated with high- and low-frequency operations. While resonance-based systems offer enhanced performance and efficiency at specific frequencies, they may be limited in their operational range. In contrast, off-resonance systems provide greater flexibility in frequency selection but may require higher energy input for comparable actuation effects. Discussing these nuances offered valuable insights into the design considerations and optimization strategies for ceria-based electromechanical devices.

In conclusion, our study provides insights into how material properties, device designs, and potential applications come together in flexible electromechanical systems using ceria actuators. By understanding these key factors, we pave the way for developing more advanced and functional flexible electronics and optoelectronics. Our findings underscore the versatility and potential of ceria-based actuators in enabling next-generation flexible electromechanical systems with enhanced functionality and performance.

## MATERIALS AND METHODS

### Sputtering deposition

CGO [10% (at.) gadolinium-doped ceria] thin films with a ≈400 nm thickness were deposited by RF-MS. The source was operated at 13.6 MHz, delivering 75 W and a DC self-bias (*V*_SB_) of 304 *V*_DC_. The depositions were carried for 1.5 hours under argon (Ar) flow at 5.10^−2^ torr, with a chamber base pressure of 1.9 × 10^−6^ torr.

Titanium nitrate (TiN) films were also deposited by sputtering, but since TiN exhibits favorable electronic conductivity, the conventional DC magnetron sputtering technique was used. TiN was used for both electrodes and the bonding layer. TiN films were deposited for 1.5 hours with a 350 *V*_DC_ and 0.2 A, rendering ≈60 W power. The deposition was performed under a 1.5 × 10^−2^ torr pressure sustained by Ar flow, with a camber base pressure of 1.4 × 10^−5^ torr.

Two electrode configurations were used to create the samples: in-plane and cross-plane electrodes. For the in-plane electrodes, multiple patches were deposited on the sample’s surface using a shadow mask, enabling the application of an electric field parallel to the surface. Cross-plane geometry comprises top and bottom electrodes separated by the active CGO thin film.

Flat FS glass, 140 μm thick, was used as a benchmark for rigid substrates. PI (75 μm) and PET (178 μm) were used to deposit the flat devices for flexible substrates. The PET was acquired precoated with a 1000-Å-thick ITO layer (Sigma-Aldrich). For the tubular devices, two different materials were used (ID, inner diameter; WT, wall thickness): FS (ID, 99 μm; and WT, 10 μm) and PI (ID, 127 μm; and WT, 19 μm). The tubular substrates were provided by companies Hilgenberg and Zeus, respectively.

Because of the small and delicate nature of the tubular devices, handling them is not as straightforward as with flat substrates. The tubes were cut into approximately 2 cm lengths and affixed within a metallic scaffold using PI adhesive tape to facilitate manipulation. This setup allows safe manipulation of the tubes and control of the side where the thin films are deposited. In addition, the metallic framework serves a dual purpose, acting as a protective barrier for the deposited films and a stable mechanical base for subsequent electromechanical measurements.

Tubular devices were created using mechanical masks to expose only the area in which deposition was desired. The masks were made from PI tubes (ID, 254 μm; and WT, 25 μm) with a slightly larger diameter than the tubes used as substrate. The masks were cut and placed around the desired areas using a low-magnification optical microscope, creating geometries in-plane and cross-plane devices.

### Microstructure and morphology

The microstructure of the thin film was visualized using a Zeiss Ultra-55 field-emission scanning electron microscope. The cross-sectional view of the flat films was acquired by fracturing or cutting the substrate to reveal the structure. Because of the complex geometry and limited dimensions, the cross-sectional view was achieved for the tubes by cutting and polishing the surface with an ion mill (E-3500 Ion Milling System).

The cross-sectional high angle annular dark field–scanning transmission electron microscopy lamellae are prepared with a focused ion beam FEI HELIOS 650 dual-beam. Protective layers of carbon and Pt were deposited on the top of the film. The analysis uses a probe aberration-corrected FEI-TITAN 80-300 electron microscope at 300 kV and equipped with the SUPER-X EDX detector.

### Crystal structure characterization

XRD patterns were acquired with a Rigaku Miniflex 600 from 5° to 110° (2θ) with a 0.005° step and 1°/min rate. Copper radiation (CuKα, 1.54 Å) filtered by a nickel foil was used. Additional in operando XRD measurements were conducted at the DanMAX synchrotron beamline at MaxIV (*67*). The samples were placed vertically in a transmission geometry, and voltage was applied using a DC power supply connected by copper wires to the sample’s top and bottom TiN electrodes. Electric bias from 0 V to 32 V was incrementally applied simultaneously to the XRD measurement, simulating an in operando condition. The x-ray radiation was set to a 15-keV energy, and the diffraction patterns were captured with a DECTRIS PILATU3 X 2M CdTe area detector.

### Electromechanical measurement

For the electromechanical characterization, the vertical displacement of the thin film was tracked using a nanovibration analyzer equipped with a single beam laser interferometer (SIOS NA Analyzer) coupled with an Ametek 7230 DSP lock-in amplifier. The lock-in amplifier does not differentiate the direction of displacement, i.e., contraction or expansion. Thus, all experimental data and calculated coefficients are absolute values. The sample is placed on an optical table to reduce vibration from the outside environment. A sinusoidal electric field was applied using an Aim-TTI TGP 3100 function generator connected to a Trek 2220 voltage amplifier. This setup allows measurements from DC to 10 kHz with an amplitude resolution of 0.1 nm.

For the flat samples, assuming the sample behaves as an ideal cantilever being homogeneously strained by a thin film, the displacement measured at a point of a can be translated into curvature using Eq. 1.

$$\Delta k=\frac{2d}{L^{2}}$$ (1)

Where Δ*k* is the curvature, *d* the measured displacement, and *L* the distance from the measured point to the fixed region of the cantilever. From the curvature (Δ*k*), the stress induced by the thin film can be calculated using Stoney’s Eq. 2 (*68*, *69*).

$$∆\sigma =\frac{Y_{s}}{1-\nu_{s}}\frac{t_{s}^{2}}{6t_{f}}∆k$$ (2)

Were *Y*_s_ and ν_s_ are the Young’s modulus and Poisson’s ratio of the substrate, *t*_s_ is the substrate’s thickness and *t*_f_ is the film thickness.

Since ceria is an electrostrictor, a slightly different methodology from classic piezoceramics is necessary to convert the stress to the electromechanical coefficients. Electrostrictors exhibit an electromechanical response proportional to the square of the electric field and at double the frequency (second harmonic) (Eq. 3). Such a feature enhances the versatility of ceria actuators since it can mimic the inverse piezoelectric effect (*10*, *45*). By applying a bias simultaneously with an alternating electric field, a response at the same frequency and linear to the electric field is induced Eq. 4.

$$\sigma \propto {\left\{U_{\text{AC}}\cdot \text{cos}\left(2\pi \text{ft}\right)+U_{\text{DC}}\right\}}^{2}$$ (3)

$$U_{\text{DC}}^{2}+\frac{U_{\text{AC}}^{2}}{2}+2U_{\text{AC}}\cdot U_{\text{DC}}\cdot \text{cos}\left(2\pi \text{ft}\right)+\frac{U_{\text{AC}}^{2}}{2}\cdot \text{cos}\left(2\cdot 2\pi \text{ft}\right)$$ (4)

Where σ is the stress, *U*_AC_ and *U*_DC_ are the alternating electric field, bias, *f* is the frequency, and *t* is the time.

From the stress calculated in Eq. 2 and the electromechanical coupling shown in Eqs. 3 and 4, the electromechanical coefficients can be calculated. From the second harmonic, we can define the electrostriction coefficient (*M_ij_*) (*4*) (Eq. 5). From the bias-induced first harmonic, we can define the so-called pseudo-piezo coefficient (*e_ij_*) (*10*) (Eq. 6).

$$M_{\mathrm{ij}}=\frac{\sigma_{j}}{Y\cdot E_{i}^{2}}$$ (5)

$$e_{\mathrm{ij}}=\frac{\sigma_{j}}{Y\cdot E_{i}}$$ (6)

Where *Y* is the Young’s modulus of the thin film (CGO ≈ 200 GPa) (*70*) and *E* is the electric field.

Stoney’s formalism was used to calculate the electromechanical coefficients of all flat samples. For the tubes actuated along their length (elongation/contraction), a simpler approximation can be assumed to calculate the coefficients for the thin films. The simple traction geometry allows a straightforward conversion between the strain measured and the force induced by the thin film on the substrate. Thus, Eq. 7 can be used to calculate the stress at the thin film, which can then be applied in Eqs. 5 and 6 to determine the electromechanical coefficients.

$$\begin{array}{c} \sigma =\frac{Y_{s}\cdot \varepsilon \cdot \pi \cdot \left[{\left(\frac{\text{ID}}{2}+\text{WT}\right)}^{2}-{\left(\frac{\text{ID}}{2}\right)}^{2}\right]}{\pi \cdot \left[{\left(\frac{\text{ID}}{2}+\text{WT}+\text{TF}\right)}^{2}-{\left(\frac{\text{ID}}{2}+\text{WT}\right)}^{2}\right]}\\ =\frac{\varepsilon \cdot Y_{s}\cdot \left(\text{ID}\cdot \text{WT}+{\text{WT}}^{2}\right)}{\text{TF}\cdot \left(\text{ID}+\text{TF}+2\text{WT}\right)} \end{array}$$ (7)

*Y*_s_ is Young’s modulus of the substrate, ε is the measured strain, ID is the internal tube diameter, WT is the tube’s wall thickness, and TF is the thin film thickness.

### Theory and numerical simulation

Our theoretical treatment builds on our previous work on piezoelectric actuation of microfluidic systems formulated as a second-order perturbation theory of the continuum fields (*71*, *72*). Here, we restrict the independent fields to be the mechanical displacement field ***u*** and the electric potential ϕ, and from these, we derive the dependent fields, such as the strain and stress tensors ***s*** and σ, as well as the electric field and electric displacement ***E*** and ***D***. As governing equations, we use Cauchy’s equation $\rho \partial_{t}^{2}u=\nabla \cdot \sigma$ and the charge-free first Maxwell equation **∇** · ***D*** = 0. The present work deviates from our previous work by including the nonlinear electrostrictive couplings in the constitutive equations, which, for a given time-harmonic driving voltage at angular frequency ω, leads to higher harmonics in the electromechanical response of the system. Using the index notation, our constitutive equations are

$$\sigma_{\mathrm{ij}}=C_{\text{ijkl}}^{E}\;s_{\mathrm{kl}}+m_{\text{ijkl}}\;E_{k}E_{l}$$ (8a)

$$D_{i}=-2\;m_{\text{ijkl}}s_{\mathrm{kl}}E_{j}+\varepsilon_{\mathrm{ij}}^{s}\;E_{j}$$ (8b)

where $\;C_{\text{ijkl}}^{E}$ are the elastic moduli, *m_ijkl_* are the electromechanical coupling constants, and $\varepsilon_{\mathrm{ij}}^{s}$ the electric permittivities. We use complex-valued phase factors and study two cases in the limit of small voltage actuation, purely time-harmonic AC voltage actuation *V*^AC^ e^*i*ω*t*^, and combined DC and AC voltage actuation *V*^DC^ + *V*^AC^ e^*i*ω*t*^ on the surface electrodes of the CGO film. For the pure AC case, the dominant electrostriction response is second-order harmonic and proportional to |*V*^AC^|^2^

$$V=V^{\left(1\right)}e^{-i\omega t}+V^{\left(1\right)*}e^{i\omega t}$$ (9a)

$$u_{i}=u_{i}^{\left(0\right)}+u_{i}^{\left(2\right)}e^{-i2\omega t}+u_{i}^{\left(2\right)*}e^{i2\omega t}$$ (9b)

Where the asterisk denotes complex conjugation. For the combined DC and AC voltage drive *V*^DC^ + *V*^AC^ e^*i*ω*t*^, the dominant electrostriction response is a first-order time-harmonic and proportional to |*V*^DC^ *V*^AC^|

$$V=V^{\left(0\right)}+V^{\left(1\right)}e^{-i\omega t}+V^{\left(1\right)*}e^{i\omega t}$$ (10a)

$$u_{i}=u_{i}^{\left(0\right)}+u_{i}^{\left(1\right)}e^{-i\omega t}+u_{i}^{\left(1\right)*}e^{i\omega t}$$ (10b)

As in our previous work, we implement these equations in the Weak Form PDE Interface of Comsol Multiphysics.

## Notes

**Correction (23 January 2025):** The authors inadvertently omitted the following acknowledgment from the original version of the article: “We acknowledge MAX IV Laboratory for time on Beamline DanMAX under Proposal 20230258. Research conducted at MAX IV, a Swedish national user facility, is supported by the Swedish Research council under contract 2018-07152, the Swedish Governmental Agency for Innovation Systems under contract 2018-04969, and Formas under contract 2019-02496.” The XML and PDF versions of the article have been updated.

## Supplementary Materials

### This PDF file includes:

Supplementary Text Figs. S1 to S9 Legend for movie S1 References
