# Supplementary material for: Oxygen-defective electrostrictors for soft electromechanics
Source: Sci Adv. 2024 Aug 30;10(35):eadq3444. doi: 10.1126/sciadv.adq3444 (PMC11364099; doi:10.1126/sciadv.adq3444)
Supplement: Supplementary file 1 — Supplementary Text Figs. S1 to S9 Legend for movie S1 References [file sciadv.adq3444_sm.pdf]

Science Advances
AAAS

Supplementary Materials for

Oxygen-defective electrostrictors for soft electromechanics

Victor Buratto Tinti *et al.*

Corresponding author: Victor Buratto Tinti, victin@dtu.dk; Vincenzo Esposito, vies@dtu.dk

Sci. Adv. **10**, eadq3444 (2024)
DOI: 10.1126/sciadv.adq3444

The PDF file includes:

Supplementary Text
Figs. S1 to S9
Legend for movie S1
References

Other Supplementary Material for this manuscript includes the following:

Movie S1

Supplementary Text

Flat ceramic depositions

Fig. S1a displays the X-ray diffraction (XRD) results for the sputtered ceramics films (TiN and CGO) on different substrates (Silica Glass, Polyimide, PET). The diffraction pattern of the deposited film matches the cubic structure of TiN ($Fm\bar{3}m$ – ICSD #152807) (*73*), with no evidence of second phases. The TiN diffraction peak appears relatively broad, especially compared to the CGO10 film, indicating a more prominent nanometric characteristic (*74*). Depositing pure and single-phase TiN can be challenging due to the high affinity between oxygen and titanium (*75*). However, no signs of oxidized species were observed under the deposition conditions.

Notably, a diffraction band between 20° to 35° and three diffraction peaks are observed for all samples deposited in PI, which can be attributed to the semicrystalline PI substrate (*76*). **Fig. S1a** also shows the XRD for the CGO10 film deposited with a TiN adhesion layer at room temperature. The diffraction pattern of the CGO10 film aligns with the expected fluorite structure of ceria ($Fm\bar{3}m$ – ICSD #241840) (*77*). No additional peaks related to a second phase were identified. Regardless of the room temperature deposition, the diffraction peaks of ceria are visible and show a relatively broad shape, indicating a nanocrystalline microstructure.

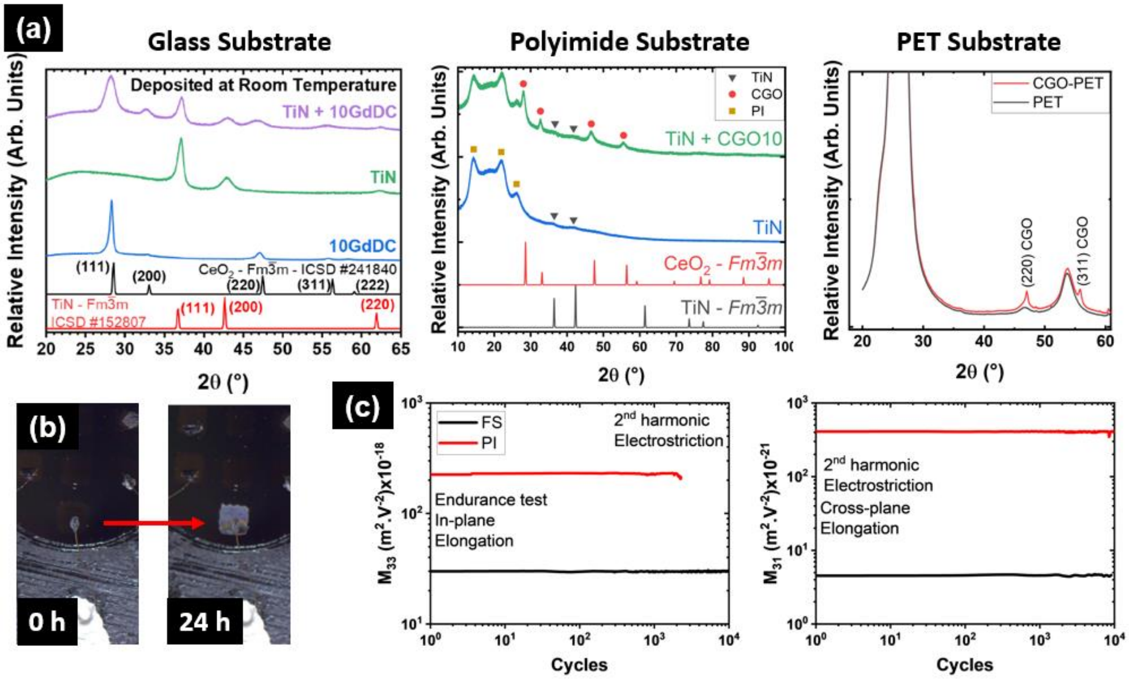

Fig. S1. Flat devices. (a)XRD patterns for the TiN and CGO films deposited on Silica Glass, Polyimide, and PET. (b) Photos of the effect of the sample left under bias (0.4 MV/cm) for 24 hours. (c) Endurance test for the electromechanical response measure for in-plane ($E_{AC} \approx 4$ kV/cm) and cross-plane ($E_{AC} \approx 40$ kV/cm) devices.

Theory and simulation

The constitutive equations [8] used for the simulations introduce the electromechanical coupling tensor $\boldsymbol{m}$ relating the stress $\boldsymbol{\sigma}$ to the electric field $\boldsymbol{E}$. Since the strain tensor $\boldsymbol{s}$ can be calculated from the compliance $(\boldsymbol{C}^E)^{-1}$ and the electromechanical coupling tensor $\boldsymbol{M}$ as

$$s_{ij} = M_{ijkl}E_kE_l + (C^E)^{-1}_{ijkl}\sigma_{kl}. \qquad \text{[S1]}$$

It can be shown by insertion into Eq. [8a] that $\boldsymbol{m}$ is related to $\boldsymbol{M}$ through $m_{ijkl} = -C^E_{ijmn}M_{mnkl}$, where $\boldsymbol{M}$ is given through its Voigt components M_{31} and M_{33} as

$$M_{ijkl} = M_{31}\delta_{ij}\delta_{kl} + \frac{1}{2}(M_{33} - M_{31})(\delta_{ik}\delta_{jl} + \delta_{il}\delta_{jk}). \qquad \text{[S2]}$$

At 10 Hz, the in-plane tube measurements and simulations indicate that $M_{33} = -6.0 \times 10^{-16}\,\frac{\mathrm{m}^2}{\mathrm{V}^2}$. We use this value to estimate M_{31} from the remaining simulations. Since M_{31} is small, it can be assumed to have an affine relation to the measured deflection u_z.

$$u_z = u_z^0 + \alpha M_{31}, \qquad \text{[S3]}$$

Where α is calculated from the simulations as $\alpha = \frac{\partial u_z}{\partial M_{31}}$. Since only the magnitude of the deflections $|u_z|$ was measured, each experiment corresponds to two possible values of M_{31} given by

$$M_{31}^+ = \frac{|u_z| - u_z^0}{\alpha}, \qquad \text{[S4a]}$$

$$M_{31}^- = \frac{-|u_z| - u_z^0}{\alpha}. \qquad \text{[S4b]}$$

Both possible values of M_{31} is plotted for each experiment in **Fig. S2**. The simulations show that the Polyimide tube experiment fails to agree with the other three experiments. This inconsistency could result from sideways vibrational modes induced in the tube, as it were freestanding under measurement. The three other measurements are consistent with $M_{31} = 0 \pm 2 \times 10^{-20}\ \mathrm{m}^2/\mathrm{V}^2$. The cantilever experiments also appear to measure M_{31} more precisely than the tubes, which can be attributed to their lower sensitivities towards M_{33}.

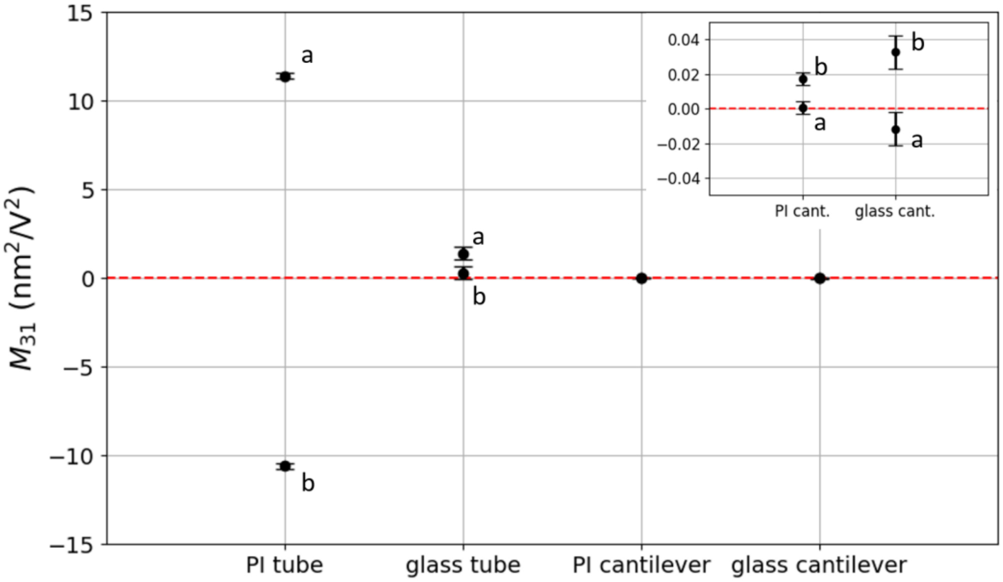

Fig. S2. Electrostriction coefficient simulation and fit. Values of $\boldsymbol{M_{31}}$ at 10 Hz fitted to match with experimental data, assuming $\boldsymbol{M_{33} = 6.0 \times 10^{-16}\ \mathrm{m^2/V^2}}$. Two possible values, a and b, of $\boldsymbol{M_{31}}$ as calculated from eqs. [S4] are plotted for each experiment.

The role of the fringe-field effect in the CGO film below the edges of the top electrode discussed in **Fig. 1(b)** in the main paper is elucidated further in **Fig. S3**. Here, the simulated deflection u_z is plotted along the center line of the glass cantilever as a function of the x-coordinate. It is seen how roughly half the deflection slope is created in the region below the side edges of the top electrode, and the remaining slope is created in a kink-like behavior below the end edge of the top electrode. Note that in this simulation, the longitudinal and transverse coefficients are set to $M_{33} = -6.0 \times 10^{-16}\ \mathrm{m^2/V^2}$ and $M_{31} = 0\ \mathrm{m^2/V^2}$.

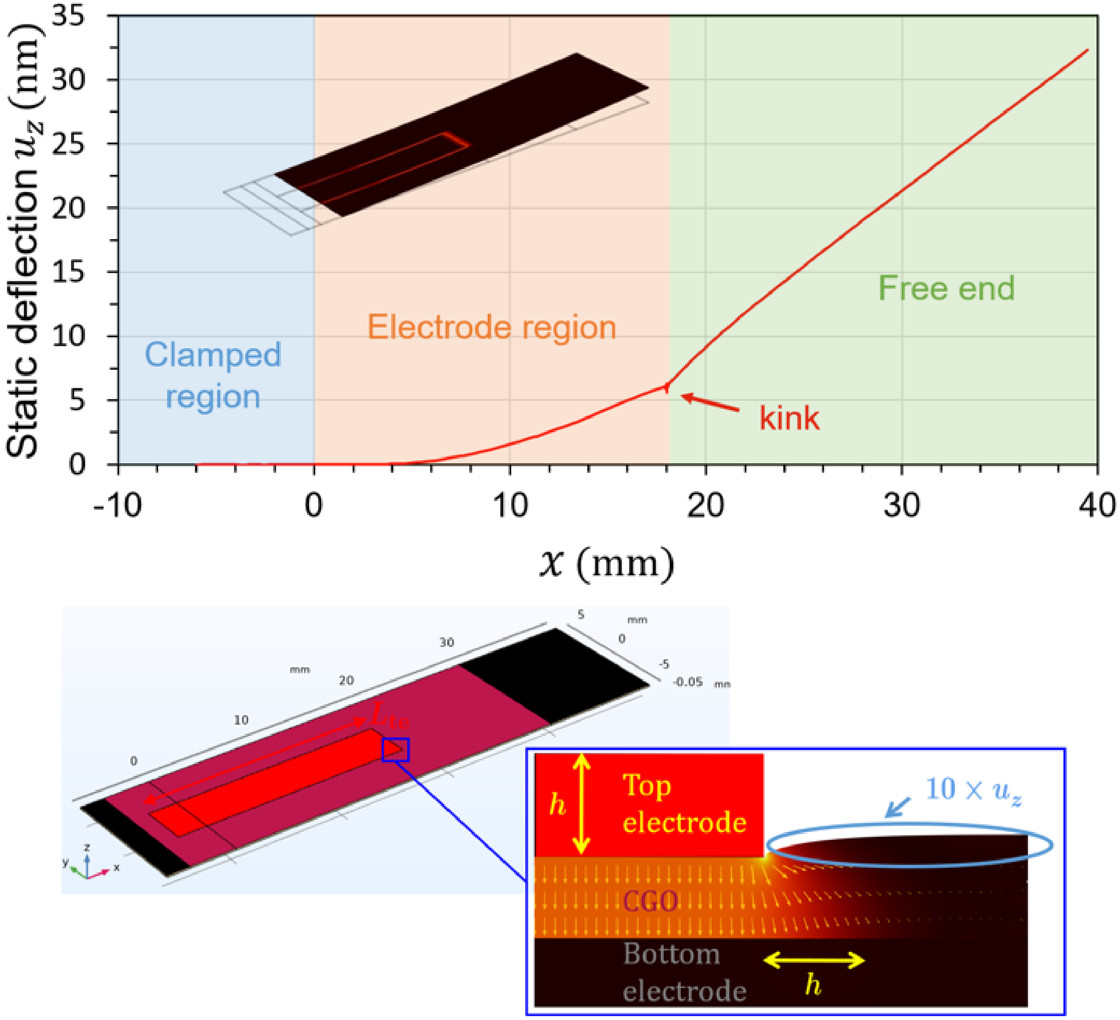

Fig. S3. Simulated cantilever deflection. Simulated deflection $\boldsymbol{u_z}$ (red curve) along the center line of the glass cantilever as a function of the $\boldsymbol{x}$-coordinate. Three regions along $\boldsymbol{x}$ are marked: the clamped region with $\boldsymbol{u_z = 0}$ (blue), the region containing the top electrode (red), and the end region without the top electrode (green). The inset shows a color plot of the magnitude $|\boldsymbol{s_{xx} + s_{yy}}|$ of the simulated static in-plane stress from zero (black) to maximum (red) induced in the substrate, clearly showing that the main contribution is generated in the regions below the edges of the top electrode. The CGO is in this simulation assumed to have electrostriction coefficients of $\boldsymbol{M_{33} = -6.0 \times 10^{-16}}$ $\mathbf{m^2/V^2}$ and $\boldsymbol{M_{31} = 0}$.

In-operando Synchrotron Transmission X-ray diffraction (*io*T-XRD)

During the 'loopscan' performed at DanMax, Max IV, multiple diffractograms were measured at the same position on the sample while steadily increasing the voltage applied to the sample (**Fig. S4**). A baseline removal operation was optionally conducted to avoid influence from the substrate or air scattering. Regions around the (111), (200), and (311) peaks were selected as regions of interest (ROIs) for tracking changes in peak positions for voltage variation. A Lorentzian fitting program using the 'curve_fit' function from the SciPy package was employed on these ROIs. The program identified parameters that provided the best fitting performance and returned the centre position of the fitted peak. By plotting the positions from each diffractogram, trends with voltage were revealed.

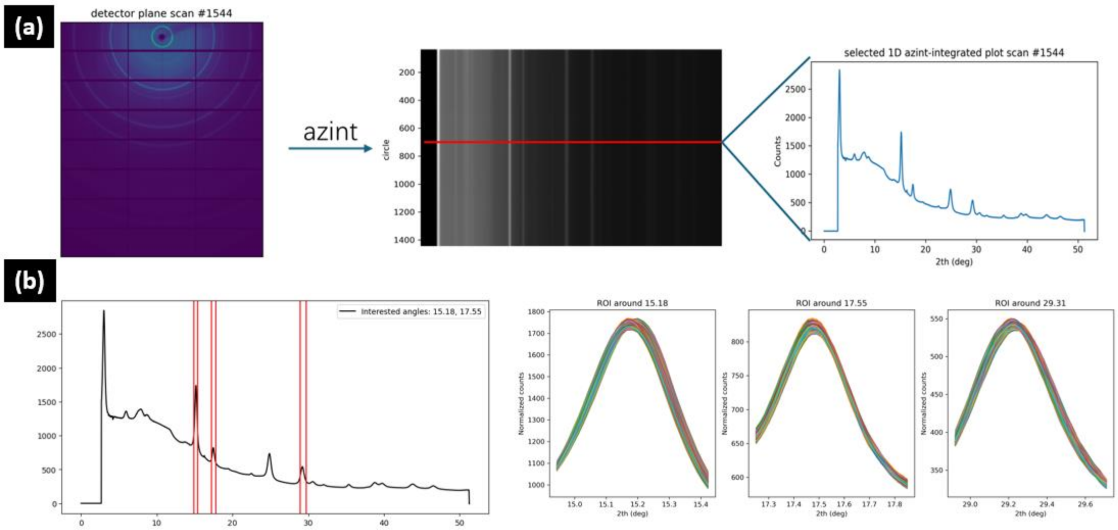

Fig. S4. *io*T-XRD measurement and analysis. (a) The X-ray diffraction patterns measured during the synchrotron experiments were captured with an area detector. A frame was taken every second with a 0.1 s exposure while an external bias was applied from 0 V to 15 V at a 0.1 V/s rate. The graphs to the right show the integrated diffraction rings stacked over the applied voltage. (b) The (111), (200), and (311) diffractions were fitted with a Lorentzian function to track the peak position in function of voltage.

Tubular depositions

A notable observation (**Fig S5**) is that the films exhibit increasing columnar defective structures as the deposition angle increases. Additionally, the inclined growth of isolated columns becomes apparent. Such effects are well-known consequences of thin film deposition at oblique angles and are caused by shadowing effects (*41*). When a deposited particle grows at an inclined angle, it creates a shadow on its side, preventing material deposition and forming inclined columns separated by crevices. Due to the directional characteristics of physical vapour deposition (PVD) techniques, such features are inevitable when depositing on static inclined surfaces, such as tubular substrates. The low-temperature deposition further contributes to forming such columnar structures due to the low atomic mobility at the surface during the deposition (*78*).

Fig. S5 shows the combined film thickness (CGO10+TiN) as a function of the deposition angle on the tube's surface. If the film thickness solely depended on the perpendicular exposed surface area, a decrease proportional to the cosine of the surface inclination ($\cos\theta$; θ: surface angle) would be expected, resulting in no film at 90°. However, a nearly linear decrease is observed, with orthogonal regions exhibiting approximately half the thickness of the top surface. Both the cosine distribution of atoms from the target and scattering effects reduce the directional nature of sputtering deposition, thereby diminishing the dependence of film thickness on the relative exposed area (*35*).

Upon closer inspection of the top of the tube (0°), all six layers are visible, with two CGO10 layers (3rd and 4th) sandwiched between four TiN layers (1st, 2nd, 5th, and 6th) (**Fig. S5**). The 180° position exhibits a more defective microstructure than the 0° position, which can be attributed to shadowing effects during film growth. Nevertheless, the porosity on the angled positions is not severe, and there is a significant volume of dense films surrounding it. Moreover, the film morphology is mirrored for the centre of the tube, resulting in a symmetric device and preventing top-bottom electrode shorts.

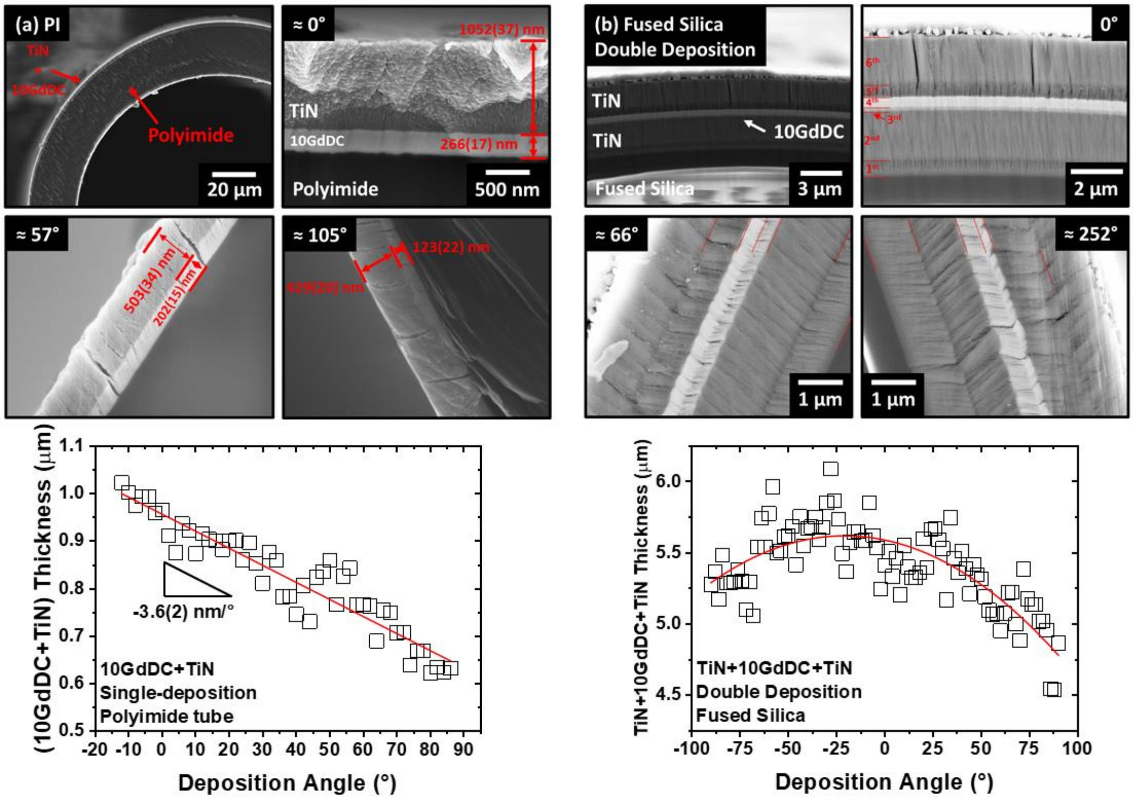

Fig. S5. Single and double deposition. Morphology and combined thickness over the inclined surfaces of the films produced with (a) single and (b) double depositions (2 depositions with 180° rotation).

Tubular Devices Electromechanical Response

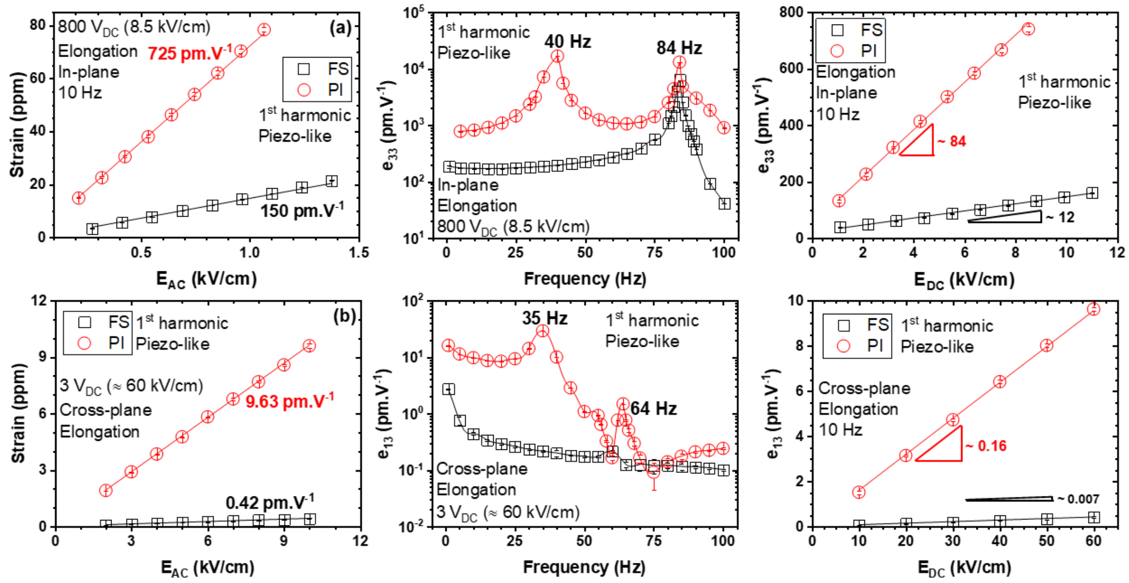

Fig. S6. Pseudo-piezoelectric performance. Bias-induced pseudo-piezo-response in the field, frequency, and bias function for the (a) in-plane and (b) cross-plane tubular devices.

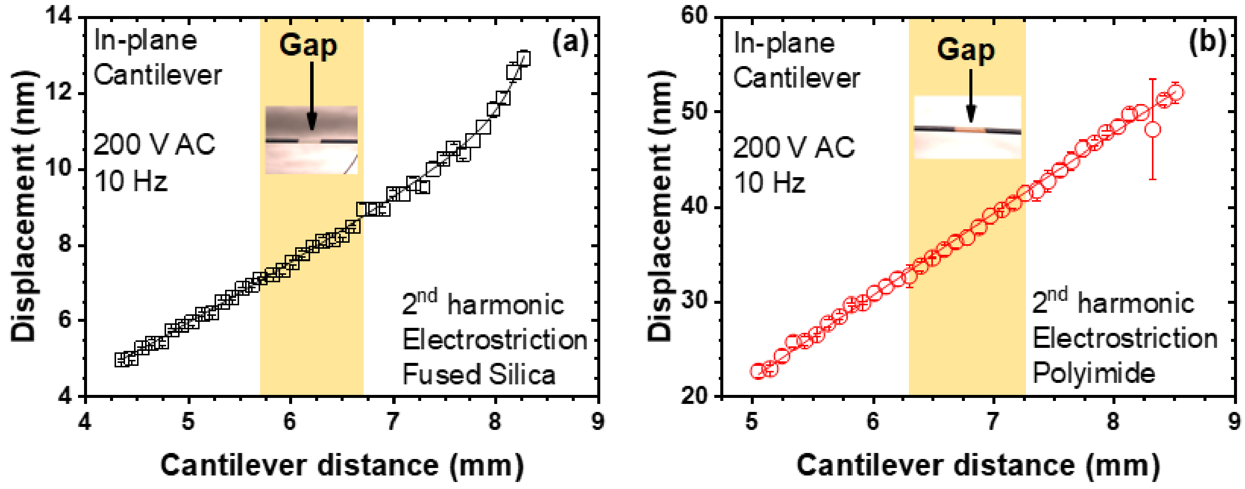

Fig. S7. Asymmetric tubes. (a) Fused silica and (b) PI tubes actuated in cantilever mode with asymmetric films.

Planar lens electromechanical response

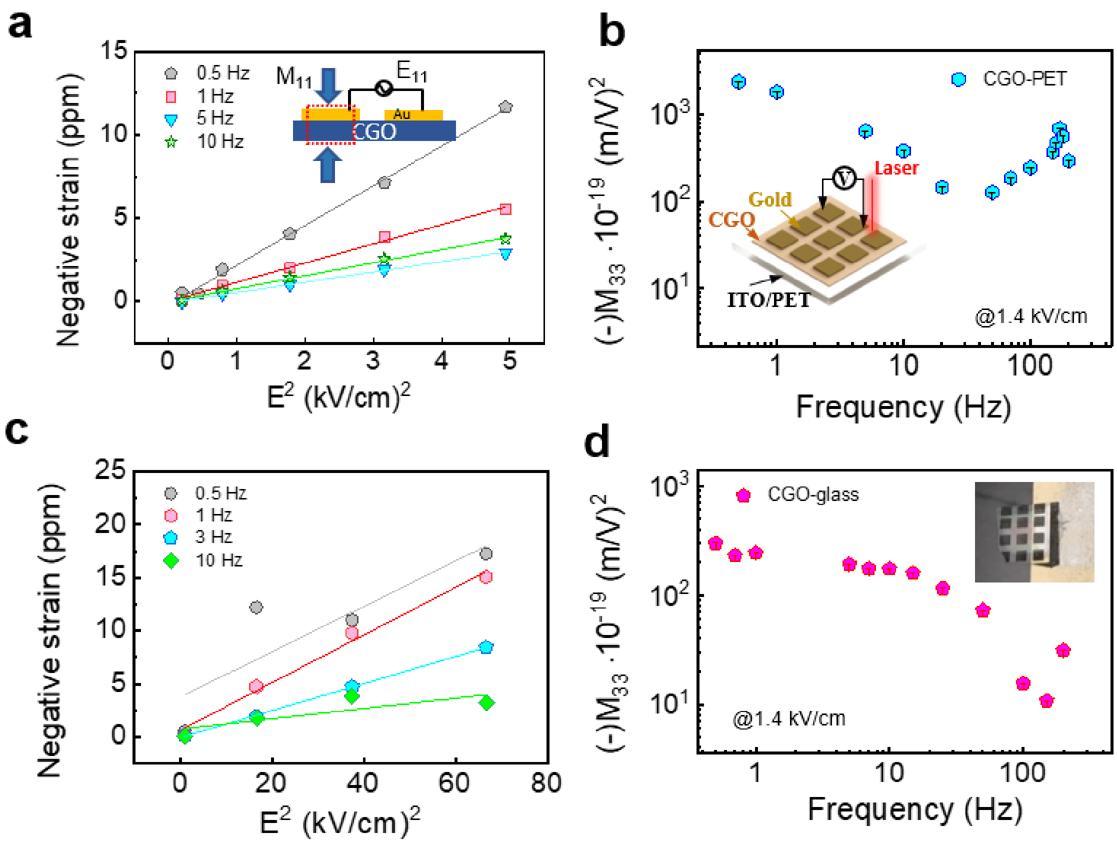

Fig. S8. In-plane transparent devices. Electromechanical response of the in-plane devices deposited on (a-b) PET and (c-d) glass.

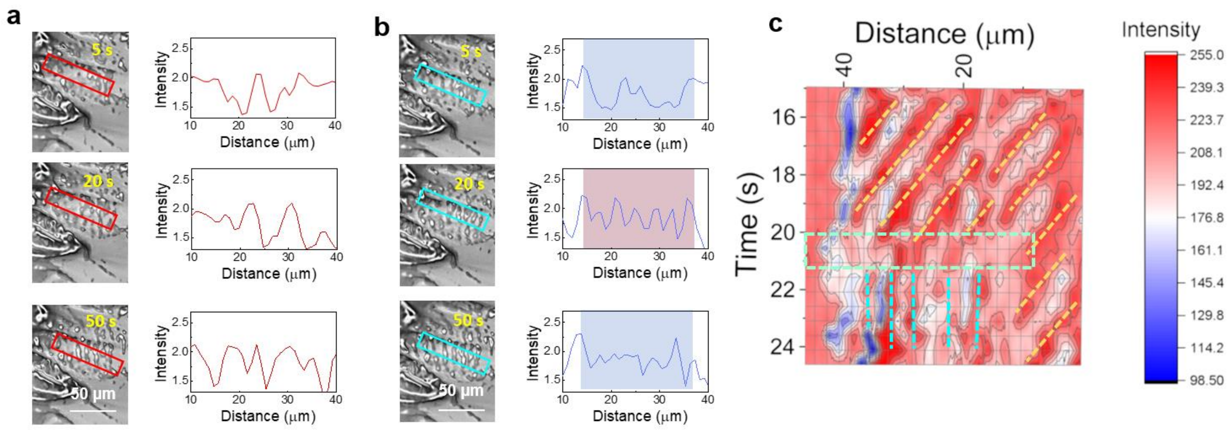

Fig. S9. Lens actuation. Variations of scattered light intensity at the lens's optical path being excited at (a) 50 Hz and (b-c) 105 Hz.

Movie S1. Lens operation. Optical microscopy view of two different regions at the central region of the proof-of-concept lens. (left) Central position close to the laser interferometer spot. (right) Position to the right of the laser spot where scattered light is visible. The device starts at the "off" state, with no external voltage. At 14s, an alternating voltage (AC) of 6 V at the resonant frequency is applied. The voltage is removed at 42 s.

REFERENCES AND NOTES

1. V. Sundar, R. E. Newnham, Electrostriction and polarization. *Ferroelectrics* **135**, 431–446 (1992).

2. R. E. Newnham, V. Sundar, R. Yimnirun, J. Su, Q. M. Zhang, Electrostriction: Nonlinear electromechanical coupling in solid dielectrics. *J. Phys. Chem. B* **101**, 10141–10150 (1997).

3. H. Uršič, M. Santo Zarnik, M. Kosec, Pb(Mg 1/3 Nb 2/3)O 3 –PbTiO 3 (PMN-PT) material for actuator applications. *Smart Mater. Res.* **2011**, 1–6 (2011).

4. R. Korobko, A. Patlolla, A. Kossoy, E. Wachtel, H. L. Tuller, A. I. Frenkel, I. Lubomirsky, Giant electrostriction in Gd-doped ceria. *Adv. Mater.* **24**, 5857–5861 (2012).

5. N. Yavo, A. D. Smith, O. Yeheskel, S. Cohen, R. Korobko, E. Wachtel, P. R. Slater, I. Lubomirsky, Large nonclassical electrostriction in (Y, Nb)-stabilized δ-Bi_2O_3. *Adv. Funct. Mater.* **26**, 1138–1142 (2016).

6. A. Kabir, B. Lemieszek, M. Varenik, V. Buratto Tinti, S. Molin, I. Lubomirsky, V. Esposito, F. Kern, Enhanced mechanical and electromechanical properties of compositionally complex zirconia $Zr_{1-x}(Gd_{1/5}Pr_{1/5}Nd_{1/5}Sm_{1/5}Y_{1/5})_x O_{2-\delta}$ ceramics. *ACS Appl. Mater. Interfaces* **16**, 12765–12772 (2024).

7. Y. Li, O. Kraynis, J. Kas, T.-C. Weng, D. Sokaras, R. Zacharowicz, I. Lubomirsky, A. I. Frenkel, Geometry of electromechanically active structures in gadolinium-doped cerium oxides. *AIP Adv.* **6**, 055320 (2016).

8. V. B. Tinti, A. Kabir, D. Zanetti de Florio, V. Esposito, "The role of dopant on the defect chemistry of metal oxides" in *Metal Oxide Defects* (Elsevier, 2023), pp. 313–353. https://linkinghub.elsevier.com/retrieve/pii/B9780323855884000106.

9. J. Grindlay, Electrostriction. *Phys. Rev.* **160**, 698–701 (1967).

10. R. Korobko, E. Wachtel, I. Lubomirsky, Cantilever resonator based on the electrostriction effect in Gd-doped ceria. *Sens. Actuators A Phys.* **201**, 73–78 (2013).

11. M. Varenik, B. Xu, J. Li, E. Gaver, E. Wachtel, D. Ehre, P. K. Routh, S. Khodorov, A. I. Frenkel, Y. Qi, I. Lubomirsky, Lead-free Zr-doped ceria ceramics with low permittivity displaying giant electrostriction. *Nat. Commun.* **14**, 7371 (2023).

12. A. Kabir, V. Buratto Tinti, M. Varenik, I. Lubomirsky, V. Esposito, Electromechanical dopant–defect interaction in acceptor-doped ceria. *Mater. Adv.* **1**, 2717–2720 (2020).

13. Y. Zuo-Guang, Handbook of advanced dielectric, piezoelectric and ferroelectric materials. *Mater. Today* **11**, 70 (2008).

14. S. Roberts, Dielectric and piezoelectric properties of barium titanate. *Phys. Rev.* **71**, 890–895 (1947).

15. E. Wachtel, A. I. Frenkel, I. Lubomirsky, Anelastic and electromechanical properties of doped and reduced ceria. *Adv. Mater.* **30**, e1707455 (2018).

16. H. Zhang, N. Pryds, D.-S. Park, N. Gauquelin, S. Santucci, D. V. Christensen, D. Jannis, D. Chezganov, D. A. Rata, A. R. Insinga, I. E. Castelli, J. Verbeeck, I. Lubomirsky, P. Muralt, D. Damjanovic, V. Esposito, Atomically engineered interfaces yield extraordinary electrostriction. *Nature* **609**, 695–700 (2022).

17. A. Zarkov, L. Mikoliunaite, A. Katelnikovas, S. Tautkus, A. Kareiva, Preparation by different methods and analytical characterization of gadolinium-doped ceria. *Chem. Pap.* **72**, 129–138 (2018).

18. R. Schmitt, A. Nenning, O. Kraynis, R. Korobko, A. I. Frenkel, I. Lubomirsky, S. M. Haile, J. L. M. Rupp, A review of defect structure and chemistry in ceria and its solid solutions. *Chem. Soc. Rev.* **49**, 554–592 (2020).

19. T. Das, J. D. Nicholas, B. W. Sheldon, Y. Qi, Anisotropic chemical strain in cubic ceria due to oxygen-vacancy-induced elastic dipoles. *Phys. Chem. Chem. Phys.* **20**, 15293–15299 (2018).

20. M. Hadad, H. Ashraf, G. Mohanty, C. Sandu, P. Muralt, Key-features in processing and microstructure for achieving giant electrostriction in gadolinium doped ceria thin films. *Acta Mater.* **118**, 1–7 (2016).

21. T. Liu, M. Wallace, S. Trolier-McKinstry, T. N. Jackson, High-temperature crystallized thin-film PZT on thin polyimide substrates. *J. Appl. Phys.* **122**, 164103 (2017).

22. L. Song, S. Glinsek, E. Defay, Toward low-temperature processing of lead zirconate titanate thin films: Advances, strategies, and applications. *Appl. Phys. Rev.* **8**, 041315 (2021).

23. F. A. Mohd Ghazali, M. N. Hasan, T. Rehman, M. Nafea, M. S. Mohamed Ali, K. Takahata, MEMS actuators for biomedical applications: A review. *J. Micromech. Microeng.* **30**, 073001 (2020).

24. L. Algieri, M. T. Todaro, F. Guido, L. Blasi, V. Mastronardi, D. Desmaële, A. Qualtieri, C. Giannini, T. Sibillano, M. De Vittorio, Piezoelectricity and biocompatibility of flexible $Sc_xAl_{(1-x)}N$ thin films for compliant MEMS transducers. *ACS Appl. Mater. Interfaces* **12**, 18660–18666 (2020)..

25. M. Akiyama, Y. Morofuji, T. Kamohara, K. Nishikubo, Y. Ooishi, M. Tsubai, O. Fukuda, N. Ueno, Preparation of oriented aluminum nitride thin films on polyimide films and piezoelectric response with high thermal stability and flexibility. *Adv. Funct. Mater.* **17**, 458–462 (2007).

26. J. A. Spechler, T. Koh, J. T. Herb, B. P. Rand, C. B. Arnold, A transparent, smooth, thermally robust, conductive polyimide for flexible electronics. *Adv. Funct. Mater.* **25**, 7428–7434 (2015).

27. E. Herth, K. Guerchouche, L. Rousseau, L. E. Calvet, C. Loyez, A biocompatible and flexible polyimide for wireless sensors. *Microsyst. Technol.* **23**, 5921–5929 (2017).

28. D.-J. Liaw, K.-L. Wang, Y.-C. Huang, K.-R. Lee, J.-Y. Lai, C.-S. Ha, Advanced polyimide materials: Syntheses, physical properties and applications. *Prog. Polym. Sci.* **37**, 907–974 (2012).

29. N.-T. Nguyen, X. Huang, T. K. Chuan, MEMS-micropumps: A review. *J. Fluids Eng.* **124**, 384–392 (2002).

30. J.-P. Ramy, M.-T. Cotte, J. P. Bolloch, R. Schnitzler, J.-J. Guena, C. Thebault, Optimization of the thick-and thin-film technologies for microwave circuits on alumina and fused silica substrates. *IEEE Trans. Microw. Theory Tech.* **26**, 814–820 (1978).

31. L. dos Santos-Gómez, J. Zamudio-García, J. M. Porras-Vázquez, E. R. Losilla, D. Marrero-López, Highly oriented and fully dense CGO films prepared by spray-pyrolysis and different precursor salts. *J. Eur. Ceram. Soc.* **40**, 3080–3088 (2020).

32. A. Kabir, H. Zhang, S. Colding-Jørgensen, S. Santucci, S. Molin, V. Esposito, Electro-chemo-mechanical properties in nanostructured Ca-doped ceria (CDC) by field assisted sintering. *Scr. Mater.* **187**, 183–187 (2020).

33. A. Kabir, S. Santucci, N. Van Nong, M. Varenik, I. Lubomirsky, R. Nigon, P. Muralt, V. Esposito, Effect of oxygen defects blocking barriers on gadolinium doped ceria (GDC) electro-chemo-mechanical properties. *Acta Mater.* **174**, 53–60 (2019).

34. P. Ayyub, R. Chandra, P. Taneja, A. K. Sharma, R. Pinto, Synthesis of nanocrystalline material by sputtering and laser ablation at low temperatures. *Appl. Phys. A Mater. Sci. Process.* **73**, 67–73 (2001).

35. A. H. Simon, "Sputter Processing" in *Handbook of Thin Film Deposition* (Elsevier, 2018), pp. 195–230. https://linkinghub.elsevier.com/retrieve/pii/B9780128123119000074.

36. M. Riester, S. Bärwulf, E. Lugscheider, H. Hilgers, Morphology of sputtered titanium nitride thin films on thermoplastic polymers. *Surf. Coat. Technol.* **116-119**, 1001–1005 (1999).

37. E. Lugscheider, S. Bärwulf, M. Riester, H. Hilgers, Magnetron sputtered titanium nitride thin films on thermoplastic polymers. *Surf. Coat. Technol.* **116–119**, 1172–1178 (1999).

38. S. Santucci, H. Zhang, S. Sanna, N. Pryds, V. Esposito, Enhanced electro-mechanical coupling of $TiN/Ce_{0.8}Gd_{0.2}O_{1.9}$ thin film electrostrictor. *APL Mater.* **7**, 071104 (2019).

39. M. S. Kabir, Z. Zhou, Z. Xie, P. Munroe, Scratch and wear resistance of hydrophobic CeO_{2-x} coatings synthesized by reactive magnetron sputtering. *Ceram. Int.* **46**, 89–97 (2020).

40. A. A. Solovyev, S. V. Rabotkin, A. V. Shipilova, I. V. Ionov, Magnetron sputtering of gadolinium-doped ceria electrolyte for intermediate temperature solid oxide fuel cells. *Int. J. Electrochem. Sci.* **14**, 575–584 (2019).

41. A. Barranco, A. Borras, A. R. Gonzalez-Elipe, A. Palmero, Perspectives on oblique angle deposition of thin films: From fundamentals to devices. *Prog. Mater. Sci.* **76**, 59–153 (2016).

42. A. D. Ushakov, N. Yavo, E. Mishuk, I. Lubomirsky, V. Y. Shur, A. L. Kholkin, Electromechanical measurements Of Gd-doped ceria thin films by laser interferometry. *KnE Mater. Sci.* **1**, 177 (2016).

43. V. B. Tinti, A. Kabir, J. K. Han, S. Molin, V. Esposito, Gigantic electro-chemo-mechanical properties of nanostructured praseodymium doped ceria. *Nanoscale* **13**, 7583–7589 (2021).

44. X. Q. Zhou, D. Y. Yu, X. Y. Shao, S. Q. Zhang, S. Wang, Research and applications of viscoelastic vibration damping materials: A review. *Compos. Struct.* **136**, 460–480 (2016).

45. D.-S. Park, M. Hadad, L. M. Riemer, R. Ignatans, D. Spirito, V. Esposito, V. Tileli, N. Gauquelin, D. Chezganov, D. Jannis, J. Verbeeck, S. Gorfman, N. Pryds, P. Muralt, D. Damjanovic, Induced giant piezoelectricity in centrosymmetric oxides. *Science* **375**, 653–657 (2022).

46. P. Gao, Z. Kang, W. Fu, W. Wang, X. Bai, E. Wang, Electrically driven redox process in cerium oxides. *J. Am. Chem. Soc.* **132**, 4197–4201 (2010).

47. S. Deshpande, S. Patil, S. V. Kuchibhatla, S. Seal, Size dependency variation in lattice parameter and valency states in nanocrystalline cerium oxide. *Appl. Phys. Lett.* **87**, 133113 (2005).

48. M. Zimmermann, H. Schmid, P. Hunziker, E. Delamarche, Capillary pumps for autonomous capillary systems. *Lab Chip* **7**, 119–125 (2007).

49. A. A. S. Bhagat, H. Bow, H. W. Hou, S. J. Tan, J. Han, C. T. Lim, Microfluidics for cell separation. *Med. Biol. Eng. Comput.* **48**, 999–1014 (2010).

50. C. Xia, N. X. Fang, 3D microfabricated bioreactor with capillaries. *Biomed. Microdevices* **11**, 1309–1315 (2009).

51. C.-H. Chen, W. Shih, H.-L. Huang, F.-C. Chiu, Temperature and thickness dependence of cerium oxide dielectric breakdown. *ECS Trans.* **28**, 421–425 (2019).

52. A. S. Algamili, M. H. M. Khir, J. O. Dennis, A. Y. Ahmed, S. S. Alabsi, S. S. Ba Hashwan, M. M. Junaid, A review of actuation and sensing mechanisms in MEMS-based sensor devices. *Nanoscale Res. Lett.* **16**, 16 (2021).

53. E. Mishuk, A. Ushakov, E. Makagon, S. R. Cohen, E. Wachtel, T. Paul, Y. Tsur, V. Y. Shur, A. Kholkin, I. Lubomirsky, Electro-chemomechanical contribution to mechanical actuation in gd-doped ceria membranes. *Adv. Mater. Interfaces* **6**, 1801592 (2019).

54. A. Achour, M. Islam, I. Ahmad, L. Le Brizoual, A. Djouadi, T. Brousse, Influence of surface chemistry and point defects in TiN based electrodes on electrochemical capacitive storage activity. *Scr. Mater.* **153**, 59–62 (2018).

55. H. Zhang, B. Wang, B. Brown, Atomic layer deposition of titanium oxide and nitride on vertically aligned carbon nanotubes for energy dense 3D microsupercapacitors. *Appl. Surf. Sci.* **521**, 146349 (2020).

56. W. Wu, Z. L. Wang, Piezotronics and piezo-phototronics for adaptive electronics and optoelectronics. *Nat. Rev. Mater.* **1**, 16031 (2016).

57. F. Krogmann, W. Mönch, H. Zappe, A MEMS-based variable micro-lens system. *J. Opt.* **8**, S330–S336 (2006).

58. H. G. B. Gowda, T. Graf, U. Wallrabe, "A One-Inch Aperture Piezoelectric Tunable Lens with Small Footprint" in *2021 21st International Conference on Solid-State Sensors, Actuators and Microsystems (Transducers)* (IEEE, 2021), pp. 427–430. https://ieeexplore.ieee.org/document/9495411/

59. C. Meng, P. C. V. Thrane, F. Ding, J. Gjessing, M. Thomaschewski, C. Wu, C. Dirdal, S. I. Bozhevolnyi, Dynamic piezoelectric MEMS-based optical metasurfaces. *Sci. Adv.* **7**, eabg5639 (2021).

60. Y. Yang, X. Du, C. Yi, J. Liu, B. Zhu, Z. Zhang, Structural, optical and electrical properties of CeO_2 thin films simultaneously prepared by anodic and cathodic electrodeposition. *Appl. Surf. Sci.* **440**, 1073–1082 (2018).

61. S. Santucci, H. Zhang, S. Sanna, N. Pryds, V. Esposito, Electro-chemo-mechanical effect in Gd-doped ceria thin films with a controlled orientation. *J. Mater. Chem. A* **8**, 14023–14030 (2020).

62. Y. B. Lee, J. K. Han, S. Noothongkaew, S. K. Kim, W. Song, S. Myung, S. S. Lee, J. Lim, S. D. Bu, K.-S. An, Toward arbitrary-direction energy harvesting through flexible piezoelectric nanogenerators using perovskite $PbTiO_3$ nanotube Arrays. *Adv. Mater.* **29**, 1604500 (2017).

63. J. A. Holt, S. Blackwell, L. Zani, C. Torres-Sanchez, Comparison of elastic properties of low-density polymeric foams determined by ultrasonic wave propagation and quasi-static mechanical testing. *Mater. Lett.* **263**, 127243 (2020).

64. M. Sinha, B. Erman, J. E. Mark, T. H. Ridgway, H. E. Jackson, Pulse propagation in end-linked poly(dimethylsiloxane) networks. *Macromolecules* **36**, 6127–6134 (2003).

65. D. Mandal, S. Banerjee, Surface acoustic wave (SAW) sensors: Physics, materials, and applications. *Sensors* **22**, 820 (2022).

66. N. Yavo, O. Yeheskel, E. Wachtel, D. Ehre, A. I. Frenkel, I. Lubomirsky, Relaxation and saturation of electrostriction in 10 mol% Gd-doped ceria ceramics. *Acta Mater.* **144**, 411–418 (2018).

67. M. R. V. Jørgensen, D. R. Sørensen, I. Kantor, S. F. Oller, DanMAX – The new materials science beamline at MAX IV. *Acta Crystallogr. Sect. A Found. Adv.* **77**, C821–C821 (2021).

68. G. G. Stoney, The tension of metallic films deposited by electrolysis. *Proc. R. Soc. A Math. Phys. Eng. Sci.* **82**, 172–175 (1909).

69. H. Liu, M. Dai, X. Tian, S. Chen, F. Dong, L. Lu, Modified Stoney formula for determining stress within thin films on large-deformation isotropic circular plates. *AIP Adv.* **11**, 125009 (2021).

70. M. Varenik, S. Cohen, E. Wachtel, A. I. Frenkel, J. C. Nino, I. Lubomirsky, Oxygen vacancy ordering and viscoelastic mechanical properties of doped ceria ceramics. *Scr. Mater.* **163**, 19–23 (2019).

71. A. G. Steckel, H. Bruus, P. Muralt, R. Matloub, Fabrication, characterization, and simulation of glass devices with AlN-thin-film transducers for excitation of ultrasound resonances. *Phys. Rev. Appl.* **16**, 014014 (2021).

72. A. G. Steckel, H. Bruus, Numerical study of bulk acoustofluidic devices driven by thin-film transducers and whole-system resonance modes. *J. Acoust. Soc. Am.* **150**, 634–645 (2021).

73. M. Hasegawa, T. Yagi, Systematic study of formation and crystal structure of 3d-transition metal nitrides synthesized in a supercritical nitrogen fluid under 10 GPa and 1800 K using diamond anvil cell and YAG laser heating. *J. Alloys Compd.* **403**, 131–142 (2005).

74. B. Akbari, M. Pirhadi Tavandashti, M. Zandrahimi, Particle size characterization of nanoparticles – A practical approach. *Iran. J. Mater. Sci. Eng.* **8**, 48–56 (2011).

75. N. K. Ponon, D. J. R. Appleby, E. Arac, P. J. King, S. Ganti, K. S. K. Kwa, A. O'Neill, Effect of deposition conditions and post deposition anneal on reactively sputtered titanium nitride thin films. *Thin Solid Films* **578**, 31–37 (2015).

76. R. Ishige, Precise structural analysis of polymer materials using synchrotron X-ray scattering and spectroscopic methods. *Polym. J.* **52**, 1013–1026 (2020).

77. C. Artini, M. Pani, M. M. Carnasciali, J. R. Plaisier, G. A. Costa, Lu-, Sm-, and Gd-Doped Ceria: A Comparative Approach to Their Structural Properties. *Inorg. Chem.* **55**, 10567–10579 (2016).

78. M. O. Jensen, M. J. Brett, Porosity engineering in glancing angle deposition thin films. *Appl. Phys. A.* **80**, 763–768 (2005).
